# Stop the bleed “ – Prehospital bleeding control in patients with multiple and/or severe injuries – A systematic review and clinical practice guideline – A systematic review and clinical practice guideline

**DOI:** 10.1007/s00068-024-02726-1

**Published:** 2025-02-05

**Authors:** H. Trentzsch, K. Goossen, B. Prediger, U. Schweigkofler, P. Hilbert-Carius, H. Hanken, D. Gümbel, B. Hossfeld, H. Lier, D. Hinck, A. J. Suda, G. Achatz, D. Bieler

**Affiliations:** 1 Institut für Notfallmedizin und Medizinmanagement (INM), LMU Klinikum, LMU München, Schillerstr. 53, 80336 Munich, Germany; 2 Institute for Research in Operative Medicine (IFOM), Witten/Herdecke University, Cologne, Germany; 3 BG Frankfurt/Main Trauma Centre, Frankfurt, Germany; 4 Department of Anaesthesiology, Intensive Care, Emergency Medicine, and Pain Therapy, Bergmannstrost BG-Hospital, Halle/Saale, Germany; 5 Department of Oral and Maxillofacial Surgery and Dentistry, Head Centre, Nord-Heidberg Asklepios Hospital, Hamburg, Germany; 6 Department of Oral and Maxillofacial Surgery, University Hospital Hamburg Eppendorf, Hamburg, Germany; 7 Department of Trauma, Reconstructive Surgery and Rehabilitation Medicine, Greifswald University Medical Centre, Greifswald, Germany; 8 Department of Trauma and Orthopaedic Surgery, BG Berlin Trauma Centre, Berlin, Germany; 9 Department of Anaesthesiology, Intensive Care Medicine, Emergency Medicine, and Pain Therapy, Centre of Emergency Medicine, HEMS Christoph 22, German Armed Forces Hospital, Ulm, Germany; 10 Department of Anaesthesiology and Intensive Care Medicine, Cologne University Hospital, Cologne, Germany; 11 Faculty of the Medical Service and Health Sciences, Bundeswehr Command and Staff College, Hamburg, Germany; 12 Centre for Orthopaedics and Trauma Surgery, University Medical Centre Mannheim, Medical Faculty Mannheim of Heidelberg University, Mannheim, Germany Theodor-Kutzer-Ufer 1-3, 67168; 13 Department of Trauma Surgery and Orthopaedics, Reconstructive and Septic Surgery, Sports Traumatology, German Armed Forces Hospital, Ulm, Germany; 14 Department of Orthopaedics, Trauma Surgery, Reconstructive Surgery, Hand Surgery, Plastic Surgery, and Burn Medicine, German Armed Forces Central Hospital, Koblenz, Germany; 15 Department for Orthopaedics and Trauma Surgery, Medical Faculty and University Hospital, Heinrich Heine University, Duesseldorf, Germany

**Keywords:** Stop the bleed, Prehospital, Haemorrhage, Bleeding control, Polytrauma guideline

## Abstract

**Purpose:**

Our aim was to develop new evidence-based and consensus-based recommendations for bleeding control in patients with multiple and/or severe injuries in the prehospital setting. This guideline topic is part of the 2022 update of the German Guideline on the Treatment of Patients with Multiple and/or Severe Injuries.

**Methods:**

MEDLINE and Embase were systematically searched until June 2021. Further literature reports were obtained from clinical experts. Randomised controlled trials, prospective cohort studies, and comparative registry studies were included if they compared interventions for bleeding control in the prehospital setting using manual pressure, haemostatic agents, tourniquets, pelvic stabilisation, or traction splints in patients with multiple and/or severe injuries. We considered patient-relevant clinical outcomes such as mortality and bleeding control. Transfusion requirements and haemodynamic stability were surrogate outcomes. Risk of bias was assessed using NICE 2012 checklists. The evidence was synthesised narratively, and expert consensus was used to develop recommendations and determine their strength.

**Results:**

Fifteen studies were identified. Interventions covered were pelvic binders (*n* = 4 studies), pressure dressings (*n* = 1), tourniquets (*n* = 6), traction splints (*n* = 1), haemostatic agents (*n* = 3), and nasal balloon catheters (*n* = 1). Fourteen new recommendations were developed. All achieved strong consensus.

**Conclusion:**

Bleeding control is the basic objective of treatment. This can be easily justified based on empirical evidence. There is, however, a lack of reliable and high-quality studies that assess and compare methods for bleeding control in patients with multiple and/or severe injuries. The guideline provides reasonable and practical recommendations (although mostly with a low grade of recommendation) and also reveals several open research questions that can hopefully be answered when the guideline is revised again.

**Supplementary Information:**

The online version contains supplementary material available at 10.1007/s00068-024-02726-1.

## Introduction

Uncontrolled loss of blood leads to death within the first few hours after severe trauma. In the early phase of severe injury, it is the second most common cause of death after severe traumatic brain injury [1–5]. In severely injured patients, exsanguination is the cause of death in approximately 30% of all cases. When unrecognised or inadequately controlled, blood loss was reported to be the most common avoidable cause of death [6–8]. Death caused by loss of blood was found to occur during the prehospital period [6, 9].

A completely new chapter of the *German Guideline on the Treatment of Patients with Multiple and/or Severe Injuries* investigates the effectiveness of widely used methods of bleeding control in the prehospital phase of care on the basis of anatomical aspects.

These methods can be different from those used in mass-casualty incidents or other life-threatening situations (mass-casualty terrorist incidents). These special situations, as well as prehospital thoracotomy with aortic occlusion [10, 11]and resuscitative endovascular balloon occlusion of the aorta (REBOA) [12, 13], which are invasive interventions to control massive bleeding, are not addressed here.

Our aim was to develop new evidence-based and consensus-based recommendations for bleeding control in patients with multiple and/or severe injuries in the prehospital setting.

## Methods

This guideline topic is part of the 2022 update of the German Guideline on the Treatment of Patients with Multiple and/or Severe Injuries [14]. The guideline is reported according to the RIGHT tool [15], the systematic review part according to the Preferred Reporting Items for Systematic Reviews and Meta-Analyses (PRISMA) 2020 reporting guideline [16]. The development and updating of recommendations followed the standard methodology set out in the guideline development handbook issued by the German Association of the Scientific Medical Societies (AWMF) [17]. All methods were defined a priori, following the methods report of the previous guideline version from July 2016 [18] with minor modifications, as detailed below. The introduction and discussion sections of this publication are translated from an abridged version of the original guideline text [14].

### PICO questions and eligibility criteria

Population, intervention, comparison, and outcome (PICO) questions were defined by an interdisciplinary group of clinical experts prior to conducting the systematic review. In addition, the participating professional societies involved in guideline development were asked to submit PICO questions. The overarching PICO question for this topic area was:

In adult patients (≥14 years) with known or suspected polytrauma and/or severe injuries and bleeding, does prehospital haemorrhage control using compression, haemostatic agents, tourniquets, pelvic stabilisation, or traction splints improve patient-relevant outcomes compared to any other intervention?

The full set of predefined PICO questions is listed in Table S1 (Online Resource 1). The study selection criteria in the PICO format are shown in Table 1.

**Table 1:** Predefined selection criteria

Population:	adult patients (≥ 14 years) with polytrauma and/or severe injuries^a,b^ and active bleeding
Intervention/ comparison^c^:	prehospital haemorrhage control using compression, haemostatic agents, tourniquets, pelvic stabilisation, traction splints, or any comparator
Outcomes:	any patient-relevant clinical outcomes, such as prevention of deaths from bleeding before arrival at a hospital, mortality (early/6 h and overall inhospital mortality), control of external bleeding, transfusion requirements, haemodynamic stability
Study type:	• comparative, prospective studies (randomised controlled trials, cohort studies) • comparative registry^d^ data (incl. case–control studies) • systematic reviews based on the above primary study types
Language:	English or German
Other inclusion criteria:	• full text of study published and accessible • study matches predefined PICO question
Exclusion criteria:	• multiple publications of the same study without additional information

^a^ Defined by an Injury Severity Score (ISS) > 15, Glasgow Coma Scale (GCS) < 9, or comparable values on other scales, or, in the prehospital setting, clinical suspicion of polytrauma/severe injury with a need for life-saving interventions

^b^ Indirect evidence from other populations was eligible for inclusion if direct evidence was unavailable

^c^ Initially, prehospital REBOA/thoracotomy was part of this topic area but was then addressed in the guideline chapter on the endovascular management of haemorrhage and vascular injuries in patients with multiple and/or severe injuries

^d^ Using the Agency for Healthcare Research and Quality (AHRQ) definition of registries[19]

### Literature search

An information specialist systematically searched for literature in MEDLINE (Ovid) and Embase (Elsevier). The search strategy was developed by the information specialist and guideline methodologist based on the predefined PICO questions. It contained index (MeSH/Emtree) and free text terms for the population and intervention. All searches were performed from database inception to 23 June 2021. Table S2 (Online Resource 1) provides details for all searches. Clinical experts were asked to submit additional relevant references.

### Study selection

Study selection was performed independently by two reviewers in a two-step process using the predefined eligibility criteria: (1) title/abstract screening of all references retrieved from database searches using Rayyan software [20] and (2) full-text screening of all articles deemed potentially relevant by at least one reviewer at the title/abstract level in Endnote (Endnote, Version: 20 [Software], Clarivate, Boston, Massachusetts, USA, https://endnote.com/). Disagreements were resolved through consensus or by consulting a third reviewer. The reasons for full-text exclusion were recorded (Table S3, Online Resource 1).

### Assessment of risk of bias and level of evidence

Two reviewers sequentially assessed the risk of bias of included studies at study level using the relevant checklists from the NICE guidelines manual 2012 [21] and assigned each study an initial level of evidence (LoE) using the Oxford Centre for Evidence-based Medicine Levels of Evidence (2009) [22]. For studies with baseline imbalance and unadjusted analyses, post-hoc secondary or subgroup analyses, indirectness of the study population, or low power and imprecision of the effect estimate, the LoE was downgraded and marked with an arrow (↓). Any disagreements were resolved through consensus or by consulting a third reviewer.

### Data extraction and data items

Data were extracted into a standardised data table by one reviewer and checked by another. A predefined data set was collected for each study, consisting of study characteristics (study type, aims, setting), patient selection criteria and baseline characteristics (age, gender, injury scores, other relevant variables), intervention and control group treatments (including important co-interventions), patient flow (number of patients included and analysed), matching/adjusting variables, and data on outcomes for any time point reported.

### Outcome measures

Outcomes were extracted as reported in the study publications. For prospective cohort studies and registry data, preference was given to data obtained after propensity-score matching or statistical adjustment for risk-modulating variables over unadjusted data.

### Synthesis of studies

The evidence was organised based on anatomical regions (trunk, pelvis, extremities, areas at the junction of the trunk and its appendages, and head/face). An interdisciplinary expert group synthesised studies narratively by aggregating data on bleeding control procedures for every region, assessing when the various procedures should be used and what options for escalation were available when a procedure was unsuccessful, and balancing benefits and adverse effects. The greatest weight was placed on the following outcomes: success of bleeding control, mortality, immediate complications, and long-term adverse effects. Clinical heterogeneity was explored by comparing inclusion criteria and patient characteristics at baseline as well as clinical differences in the interventions and co-interventions.

### Development and updating of recommendations

For each PICO question, new recommendations were developed based on sufficient available evidence and/or expert consensus (labelled as “new”). An interdisciplinary expert group of clinicians with expertise in trauma and acute care reviewed the body of evidence, drafted recommendations based on the homogeneity of clinical characteristics and outcomes, the balance between benefits and harms as well as their clinical expertise, and proposed grades of recommendation (Table 2). Studies that were conducted in a military setting were included but were not used to develop recommendations except for topical haemostatic agents. In the absence of eligible evidence, good practice recommendations were made based on clinical experience, data from studies with a lower level of evidence, and expert consensus in cases where the Guideline Group felt a statement was required due to the importance of the topic. These were not graded, and instead labelled as good (clinical) practice points (GPP). For GPPs, the strength of a recommendation is conveyed via the wording, as shown in Table 2.

**Table 2:** Grading of recommendations

Symbol	Grade of recommendation	Description	Wording (examples)
⇑⇑	A	strong recommendation	“use …”, “do not use …”
⇑	B	recommendation	“should use …”, “should not use …”
⇔	0	open recommendation	“consider using …”, “… can be considered”

### Consensus process

The Guideline Group finalised the recommendations during web-based, structured consensus conferences on 13 September 2021, 26 January 2022 and 15 March 2022 via Zoom (Zoom, Version: 5 [Software], Zoom Video Communications, Inc., San José, California, USA, https://zoom.us). A neutral moderator facilitated the consensus conference. Voting members of the Guideline Group were delegates of all participating professional organisations, including clinicians, emergency medical services personnel and nurses, while guideline methodologists attended in a supporting role. Members with a moderate, thematically relevant conflict of interest abstained from voting on recommendations. Members with a high, relevant conflict of interest were not permitted to vote or participate in the discussion. Attempts to recruit patient representatives were unsuccessful. A member of the expert group presented recommendations. Following discussion, the Guideline Group refined the wording of the recommendations and modified the grade of recommendation as needed. Agreement with both the wording and the grade of recommendation was assessed by anonymous online voting using the survey function of Zoom. Abstentions were subtracted from the denominator of the agreement rate. Consensus strength was classified as shown in Table 3.

**Table 3:** Classification of consensus strength

Description	Agreement rate
strong consensus	> 95% of participants
consensus	> 75 to 95% of participants
majority approval	> 50 to 75% of participants
no approval	< 50% of participants

Recommendations were accepted if they reached consensus or strong consensus. For consensus recommendations with ≤ 95% agreement, diverging views by members of the Guideline Group were detailed in the background texts. Recommendations with majority approval were returned to the expert group for revision and further discussion at a subsequent consensus conference. Recommendations without approval were considered rejected.

### External review

During a four-week consultation phase, the recommendations and background texts were submitted to all participating professional organisations for review. Comments were collected using a structured review form. The results were then assessed, discussed and incorporated into the text by the guideline coordinator with the relevant author group.

The guideline was adopted by the executive board of the German Trauma Society on 17 January 2023.

### Quality assurance

The guideline recommendations were reviewed for consistency between guideline topic areas by the steering group. Where necessary, changes were made in collaboration with the clinical leads for all topic areas concerned. The final guideline document was checked for errors by the guideline chair and methodologist.

## Results

**Fig. 1:**
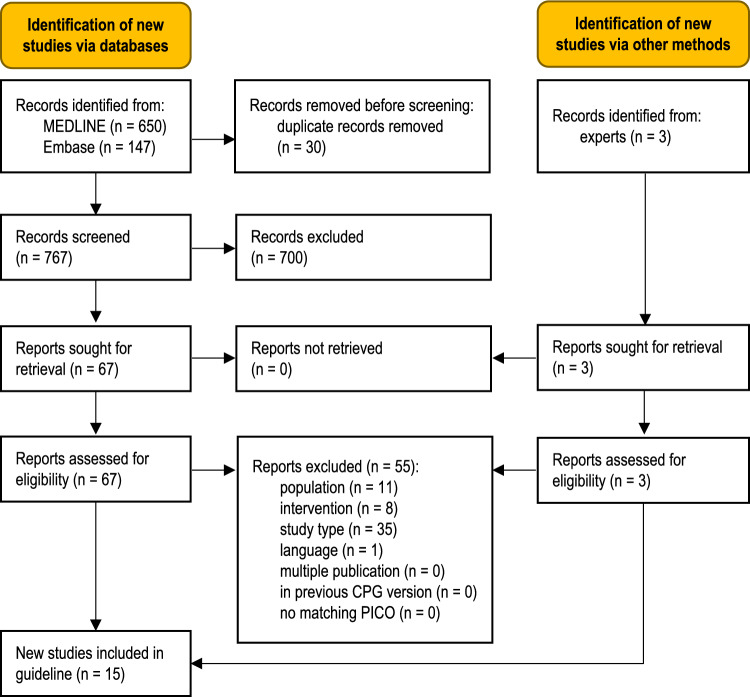
Modified PRISMA 2020 flow diagram showing the systematic literature search and selection of studies

The database searches identified 767 unique records (Fig. 1). Additional records were obtained from clinical experts. Fifteen studies were eligible for this guideline [23–25, 27–37, 69]. A total of 55 full-text articles were excluded (Table S3, Online Resource 1).

### Characteristics of studies included in this guideline

Study characteristics, main outcomes, levels of evidence, and risk-of-bias assessments are presented in Table 4. Full details are provided in Table S4, Online Resource 1. The evidence included three RCTs or quasi-experimental studies [24, 33, 34], four prospective cohort studies [27, 35, 37, 69], and eight comparative registry studies [23, 25, 28–32, 36]. Three studies were performed in North America, four in Europe, three in Asia, and five were performed in a military setting in Afghanistan or Iraq. Eligible patient populations were adults with severe injuries and known or suspected severe bleeding. Some studies were limited to subpopulations, such as patients with pelvic injuries [23–25, 69], limb trauma [28–33], scalp wounds [35], or epistaxis [37].

**Table 4:** Characteristics of studies included in the guideline (see Table S4, Online Resource 1 for details)

Study, ref, design	Population	Interventions (N patients)	Main outcomes (selection)*	LoE, risk of bias (RoB)^§^, comments
*Pelvic binders*				
Berger-Groch 2021 [23] registry study	Severely injured trauma patients with pelvic fractures^a^	PH-PCCD (N = 284) ED-PCCD (N = 170) CG: no PCCD (N = 649)	Multivariate regression Inhospital mortality, OR (95% CI) PH-PCCD: 1.49 (0.80–2.78) ED-PCCD: 1.45 (0.71–2.97)	LoE: 2b High risk of selection bias Mortality adjusted for baseline imbalance
Pierrie 2021 [24] RCT	High-energy traumatic injury, pelvic ring injury, or hypotension	IG: pelvic binder (N = 20) CG: standard of care (N = 23)	30d mortality, n/N (%) 0/20 (0) vs. 1/23 (2.3), p = 0.99	LoE: 2b↓ High risk of performance bias Underpowered for mortality (imprecision)
Pizanis 2013 [25] registry study	Patients with fractures or disruptions of the pelvic ring	Sheet: circumferential sheets (N = 31) Binder: circumferential binders (N = 28) C-clamp: c-clamps (N = 133)	Multivariate regression Mortality, OR (95% CI) Sheet: 3.26 (1.15–9.26) C-clamp: reference	LoE: 2b Unclear RoB Timing of stabilisation unclear; adj. mortality for circumferential binders n.r
Schweigkofler 2021 [69] prospective cohort study	Patients with suspected pelvic injuries	IG: prehospital pelvic binder (N = 37) CG: no pelvic binder (N = 27)	RISC-II adjusted standardisation Mortality, SMR 1.06 vs. 1.35, p = 0.50	LoE: 3b↓ High risk of selection bias Post-hoc subgroup analysis
*Pressure dressings*				
Taghavi 2021 [27] prospective cohort study	Penetrating trauma patients	IG: pressure dressing (N = 409) applied on scene (N = 325) or during transport (N = 161) CG: no prehospital procedures (N = 898)	Multivariate regression Inhospital mortality, OR (95% CI) IG: 0.80 (0.34–1.87)	LoE: 2b High risk of selection bias No information on baseline characteristics
*Tourniquets*				
Clasper 2009 [28] registry study	Military patients, lower-limb injury with fracture^b^	IG: tourniquet (N = 22 limbs) CG: no tourniquet (N = 22 limbs)	Matched cohort analysis Major complications, n/N limbs 10/22 vs. 4/22, p = 0.045 Failed salvage [amputation], n/N 3/22 vs. 3/22, n.s	LoE: 3b↓ High risk of selection and performance bias 57% of patients not severely injured (indirectness)
Henry 2021 [29] registry study	Patients with peripheral arterial injuries	IG: prehospital tourniquet (N = 97) CG: no prehospital tourniquet (N = 847)	Multivariate regression Inhospital mortality, OR (95% CI) 0.32 (0.16–0.85) Delayed amputation, OR (95% CI) 1.07 (0.21 to 10.88)	LoE: 2b High risk of selection and performance bias Patients did not meet polytrauma^e^ criteria
Kauvar 2018 [30] registry study	Military patients with at least one lower-extremity arterial injury^c^	IG: tourniquet (N = 254 limbs) CG: no tourniquet (N = 201 limbs)	Mortality, n (%) 8 (3.2) vs. 8 (4.0), n.s Amputation, n (%) 63 (25) vs. 40 (19), n.s	LoE: 2b High risk of selection and performance bias Unadj. for baseline imbalance
Kragh 2015 [31] registry study	Military patients, major limb trauma^d^	IG: tourniquet (N = 1272) CG: no tourniquet (N = 3025)	Mortality, n (%) 102 (8.0) vs. 112 (3.7), p < 0.0001	LoE: 3b↓ High risk of selection bias Unadj. for baseline imbalance, prehospital deaths excluded
Kragh 2015 [32] registry study	Military patients, major limb trauma, AIS > 2	IG: tourniquet (N = 251) CG: no tourniquet (N = 251)	Matched cohort analysis Mortality, OR (95% CI) OR 0.92 (0.45–1.87)	LoE: 2b High risk of selection bias Prehospital deaths excluded
Taghavi 2021 [27] prospective cohort study	Penetrating trauma patients	IG: tourniquet (N = 108) applied on scene (N = 86) and/or during transport (N = 29) CG: no prehospital procedures (N = 898)	Multivariate regression Inhospital mortality, OR (95% CI) IG: 0.70 (0.14–3.93)	LoE: 2b High risk of selection bias No information on baseline characteristics
*Traction splints*				
Irajpour 2012 [33] prospective, quasi-experimental study	Patients with femur fractures	IG: traction splint (N = 32) CG: simple splint (N = 32)	Pain intensity 1 h after splinting [VAS], mean ± SD 4.8 ± 1.0 vs. 6.0 ± 1.3, p = 0.0001	LoE: 2b↓ High risk of selection and performance bias No polytrauma^e^ population (indirectness)
*Haemostatic agents*				
Hatamabadi 2015 [34] RCT	Penetrating trauma patients	IG: chitosan-coated gauze (N = 80) CG: conventional gauze (N = 80)	Time to bleeding control, n (%) < *5 min*: 41 (61.2) vs. 26 (38.8) *5–10 min*: 20 (47.6) vs. 22 (52.4) ≥ *10 min*: 19 (37.3) vs. 32 (62.8) p = 0.010	LoE: 2b↓ High risk of performance bias No polytrauma^e^ population (indirectness)
Kabeer 2019 [35] prospective cohort study	Patients with bleeding scalp wounds	IG: chitosan-coated gauze (N = 47) CG: conventional cotton gauze (N = 57)	Time to haemostasis [min], mean ± SD 4.68 ± 1.04 vs. 18.56 ± 5.04, p < 0.0001	LoE: 3b↓ High risk of selection, performance and detection bias No polytrauma^e^ population (indirectness)
Winstanley 2019 [36] registry study	Military patients, major trauma	IG: haemostatic agent (N = 317) CG: no haemostatic agent (N = 3475)	Survival, % 71.3 vs. 64.0, p = 0.01	LoE: 2b High risk of selection bias Analysis unadj. for baseline confounders
*Nasal balloon catheters*				
García Callejo 2010 [37] prospective cohort study	Posterior epistaxis requiring nasal packing	IG: pneumatic packing (105 packings in N = 96 patients) CG: conventional posterior packing (47 packings in 44 patients)	Control with a single packing 71 (67.6%) vs. 37 (78.7%), p < 0.001 Pain during placement [VAS], mean ± SD 6.7 ± 1.7 vs. 8.3 ± 1.5, p < 0.001	LoE: 3b↓ High risk of selection and performance bias Analysis unadj. for baseline confounders; < 10% trauma patients (indirectness)

^*^ Data for IG versus CG unless otherwise specified; for OR, the CG is the reference group unless otherwise specified. ^§^ Risk of bias: low RoB = RoB low for all domains; unclear RoB = RoB unclear for at least one domain, no high RoB in any domain; for studies with high RoB, all domains with high RoB are named, with RoB low or unclear for all other domains (for full details Table S4, Online Resource 1). ^a^ AIS score 3–5, unstable fractures with or without relevant blood loss or open fracture, ISS ≥ 9. ^b^ AIS > 1 in the lower limbs. ^c^ Injury to the common, superficial or deep femoral, popliteal, or tibial arteries. ^d^ Extremity AIS ≥ 3, extremity AIS 1 to 5 if paired with an associated external AIS ≥ 3. ^e^ ISS ≥ 15, multiple injuries. For abbreviations and acronyms see list included

### Risk-of-bias assessment for included studies and levels of evidence

The risk of bias was unclear for one study that reported insufficient study details. No study was judged to be of low risk of bias in all domains. The risk of selection bias was high in thirteen studies, eight were at high risk of performance bias, and one was at high risk of detection bias.

The level of evidence was downgraded for eight studies. Reasons for downgrading were baseline imbalance and unadjusted analysis (one study), post-hoc subgroup analysis (one study), low power and imprecision of the effect estimate (one study), and indirectness for five studies that included patients with non-severe injuries.

### Recommendations

Nine recommendations and six good practice points were developed based on the evidence and expert consensus (Table 5). All achieved strong consensus.

**Table 5:** List of recommendations with grade of recommendation and strength of consensus

No	GoR	Evidence, consensus	Recommendations
*The fundamental concept*			
1	GPP	– 100%	Always stop active bleeding if the bleeding site can be accessed for bleeding control in the prehospital setting
*Pelvic injuries*			
2	GPP	– 100%	Clinically examine the pelvis during the prehospital phase
3	B ⇑	[69] 100%	During the clinical assessment, attention should be paid to the presence of spontaneous pain, pain on gentle palpation, and visible external injuries that provide indirect evidence of a pelvic ring injury
4	GPP	– 100%	Patients with clinical signs and symptoms of a pelvic ring injury or an unstable pelvic ring injury and haemodynamic instability should be managed with a pelvic binder
*Extremity injuries*			
5	A ⇑⇑	[27–29] 100%	Control active external bleeding from the extremities using the following stepwise approach: 1) Manual pressure 2) Pressure dressing, if possible in combination with a haemostatic agent 3) Tourniquet
*Pressure dressings*			
6	GPP	– 100%	If other options for bleeding control are available, manual pressure may be discontinued, even if it provides sufficient control, and another technique may be used instead. Repeated assessments of whether bleeding has stopped should not be performed when manual pressure is applied
7	B ⇑	[27] 100%	Pressure dressings should be applied to manage external bleeding from torso and/or extremity injuries after penetrating trauma
8	GPP	– 100%	Likewise, apply pressure dressings to manage acute bleeding from the torso and/or extremities after blunt trauma
*Tourniquets*			
9	A ⇑⇑	[27, 29] 95.5%	Use a tourniquet if life-threatening bleeding cannot be stopped in a timely manner using other methods
10	GPP	– 100%	If a tourniquet was applied for initial bleeding control at an inaccessible bleeding site, the continued use of a tourniquet or conversion to another technique should be critically assessed as soon as the patient has been rescued and the situation allows
*Haemostatic agents*			
11	A ⇑⇑	[34] 95.5%	In the case of bleeding stab wounds in which the foreign body has already been removed and which are at least 3 cm long, direct wound tamponade with Chitosan should be performed
12	B ⇑	[36] 95.5%	Gunshot and blast injuries with active bleeding should be managed with chitosan-based dressings
13	0 ⇔	[36] 100%	To support the stepwise approach to bleeding control, haemostatic agents can be used as an adjunct at all steps
*Head/facial injuries*			
14	B ⇑	[35] 95.5%	Chitosan-based dressings should be used for scalp wounds with active bleeding in order to achieve more rapid and more effective bleeding control
*Epistaxis*			
14	0 ⇔	[37] 100%	Pneumatic packing, as an alternative to posterior packing, can be used to control bleeding from the upper midfacial and/or nasal regions

GoR Grade of recommendation, GPP Good (clinical) practice point

## Discussion

### The reasons and rationale for bleeding control

#### (refers to recommendation 1)

Blood loss not only leads to hypovolaemic shock, which in itself is a life-threatening condition, but also activates the inflammatory response and the coagulation cascade. This results in the consumption of coagulation factors and may cause hyperfibrinolysis. Prolonged blood loss and haemorrhagic shock are essential factors in the development of acute trauma-induced coagulopathy. Shock is defined as inadequate tissue oxygenation as a result of hypoperfusion and a decrease in the oxygen-carrying and oxygen-delivery capacity of blood. This leads to a reduction in aerobic metabolism, an increase in the formation of lactate, and an increase in the consumption of buffer bases. Haemorrhagic shock contributes to the lethal triad of (lactic) acidosis, coagulopathy and hypothermia. The underlying processes are described in detail elsewhere [38–45]. All these factors are associated with increased mortality and morbidity as a result of organ dysfunction and multi-organ failure.

Exsanguination and haemorrhagic shock are dynamic processes. Mortality increases with the duration of ongoing uncontrolled bleeding. For example, the probability of death was reported to increase by approximately 1% for every three minutes during the first 90 min that patients with intra-abdominal bleeding spent in the emergency department [46]. For this reason, bleeding should be stopped as soon as possible.

The pivotal role of the immediate control of external catastrophic haemorrhage was addressed for the first time in the military setting where bleeding from limb wounds was found to account for most preventable deaths in patients with blast and ballistic injuries and thus suggested the need for a change in treatment priorities (< C > ABC) [47]. Today, the civilian emergency medical services too recommend a shift towards prioritising the control of catastrophic or exsanguinating haemorrhage (cABCDE or xABCDE [44, 48]). This type of bleeding usually originates from arteries of the extremities but can also occur at the scalp or at the junction of the trunk and the neck or limbs [49].

All other types of bleeding are addressed when circulation (C) is evaluated according to the xABCDE approach. Haemorrhage can be divided into external and internal (or occult) haemorrhage.

External haemorrhage is usually easy to identify. However, it is important to emphasize that external bleeding can remain undetected due to inadequate exposure. When looking for sources of bleeding, remove clothing, look under blankets, etc.

Occult haemorrhage, which refers to internal bleeding into a body cavity, is far more difficult to identify if ultra sound isn´t available in the pre-hospital setting. The four major areas of internal bleeding are the chest, abdomen, pelvis/retroperitoneum, and the proximal long bones, especially the femur [49, 50]. Bleeding into body cavities may not be apparent in the prehospital phase and is often inaccessible for interventions.

Less well known is occult bleeding associated with closed soft-tissue injuries (non-cavitary haemorrhage), e.g. degloving injuries caused by the separation of the skin and subcutaneous tissue from the underlying fascia (Morel-Lavallée lesion, for example in the pelvic region), as a result of which cavities are created and can be filled with large volumes of blood. Also reported were severe bleeding in the female breast after blunt breast trauma, corona mortis artery avulsion in association with simple fractures of the ischium/pubic ramus after low-energy injuries especially in elderly patients, bleeding into the rectus sheath after blunt abdominal trauma, and severe bleeding from the deep circumflex iliac artery after severe trauma in the inguinal region or the lateral abdominal region [51]. In addition, the site of bleeding may not be readily accessible or may not be visible in trapped patients.

In the prehospital phase of care, it is almost impossible to perform interventions that specifically address occult bleeding and require an operating room environment. Patients in this situation benefit most from rapid transport to a hospital with appropriate surgical resources.

There are no comparative studies on this issue which define what needs to be done to control bleeding in the prehospital setting and which meet the inclusion criteria of this guideline. From the clinical perspective, there is no rational alternative to bleeding control in the prehospital phase. For this reason, the experts covered this issue in a GPP and recommend that active bleeding always be stopped if the bleeding site is accessible in the prehospital setting.

The European guideline on the management of major bleeding and coagulopathy following trauma recommends that the time between injury and bleeding control be minimised. The rationale behind this recommendation is that this is an acknowledged principle in trauma care despite a lack of high-quality RCTs to support this practice. The European guideline also recommends that patients with an obvious bleeding source and patients presenting with haemorrhagic shock in extremis and a suspected source of bleeding undergo an immediate bleeding control procedure. Again, there is a lack of high-quality evidence to support this recommendation. Both key recommendations, however, were graded as strong (Grade 1a and Grade 1c, respectively) in the European guideline [52].

### Pelvis

(refers to recommendations 2 and 3)

Most pelvic ring injuries are uncomplicated and therefore easy to manage [53]. By contrast, complex pelvic trauma is potentially life-threatening and is associated with a mortality rate of up to 20% [54]. It is defined as a pelvic fracture that is complicated by local pelvic injuries to vessels, nerves, soft tissues and internal organs in the lesser pelvis [55] Depending on the type of fracture and the extent of vascular injuries, major life-threatening internal haemorrhage can occur. Haemodynamically and mechanically unstable pelvic ring injuries represent a subgroup of their own. They are associated with exsanguinating haemorrhage that complicates the course within the first 24 h and therefore require a more challenging primary treatment approach [53].

Mechanically unstable pelvic fractures are the result of anteroposterior compression (open-book injuries, Tile Type B injuries), lateral compression (lateral compression fractures, Tile Type B2/B3 fractures), or vertical shear (vertical shear fractures, Tile Type C injuries). Although different levels of bleeding have been described for the various types of fractures [56, 57], these fractures are not always associated with a relevant bleeding component. A fracture classification based on morphological features alone is thus insufficient to identify patients with severe haemorrhage.

Physical examination can provide indirect evidence of a pelvic ring injury, e.g. spontaneous pain, pain on gentle palpation, and visible external injuries such as haematoma, wounds, visible deformity, leg length discrepancy, rotational deformity of a leg, tenderness to palpation of the pubic symphysis with or without a palpable gap, abrasions, open fractures, perineal ecchymosis, and bleeding from the urethra, vagina or anus. A systematic review investigating the detection of pelvic fractures in alert and assessable patients showed that a clinical examination detects relevant pelvic fractures as accurately as conventional radiography [58]. Injuries to the pelvic ring, however, often remain undetected in the prehospital phase [59–61].

A physical examination includes an assessment of mechanical stability. The evaluation of stability is performed by gently compressing the pelvis from the sides. If there is palpable instability, diagnosis is made and the manoeuvre is discontinued. If palpation from the sides suggests pelvic ring stability, gentle compression may then be applied at the level of both anterior superior iliac spines in an attempt to apply anteroposterior pressure and to open the pelvis outwards. This manoeuvre is a subject of controversy. The sensitivity of this test is reported to be low, ranging between 26.5% and 59% [60, 62, 63]. Moreover, the injured area is subject to mechanical stress during the procedure. For this reason, mechanical stability testing, in particular repeated testing, is often discouraged, especially when other clinical evidence is available. The authors, however, are not aware of any studies reporting that mechanical stability testing led to the recurrence of bleeding after initial haemorrhage control or influenced patient survival.

The manoeuvre, however, appears to play an important role in identifying patients with an increased risk of haemorrhage. A study by Pehle et al. showed that palpable instability rather than a radiological classification was indicative of bleeding complications. Patients with palpable instability during mechanical stability testing had higher transfusion requirements, a higher rate of emergency surgical procedures, and a higher mortality rate [62].

As a result of the high risks associated with complex pelvic fractures, it can be postulated that, even in the absence of sufficient palpatory evidence of pelvic instability, the application of a pelvic binder is appropriate when there is sufficient clinical suspicion and the patient is haemodynamically unstable.

A diagnosis is suspected not only on the basis of indirect evidence from a clinical examination but also on the basis of the kinematics of an incident. Specific mechanisms of injury (high-energy motor vehicle or motorcycle accidents, falls from a height of more than three metres) combined with two indirect clinical signs are associated with a 50% probability of an unstable pelvic fracture [60]. This means that mechanical stability testing is not necessarily required if the presence of a pelvic ring injury can be suspected on the basis of the kinematics of the incident and examination results.

(refers to recommendation 4)

Knops et al. described the use and effectiveness of pelvic binders [64]. The use of these devices is thought to decrease pelvic and retroperitoneal volume and limit haemorrhage by self-tamponade.

It should be noted that not all pelvic fractures are associated with relevant bleeding [62, 65]. If haemorrhage is present, it appears reasonable to initiate control measures as early as possible. Not all types of bleeding can be managed by external mechanical stabilisation of the pelvic ring [53].

According to the clinical experience of the expert members of the author group, emergency stabilisation of a fractured pelvis can promptly control haemodynamic instability. Already in the prehospital phase of care, patients with clear clinical evidence of a mechanically unstable pelvic ring injury and haemodynamic instability must therefore be managed with non-invasive external stabilisation. There is no high-quality evidence to support this approach and comparative studies are unlikely to ever be available.

It must be assumed that only a small proportion of all bony injuries to the pelvis with mechanical instability of the pelvic ring are complex pelvic ring injuries which are associated with relevant bleeding and benefit from non-invasive pelvic stabilisation. In a review of the literature, Gänsslen et al. found that life-threatening haemorrhage occurred in only about 1–2% of all pelvic fractures [53]. The outstanding benefit of the measure experienced in individual cases can therefore presumably only be demonstrated in highly selected subgroups.

The diagnosis of an unstable pelvic ring injury is difficult to make especially in the prehospital setting as a result of the non-availability of appropriate imaging modalities. In addition, current evidence suggests that an unstable pelvic ring injury cannot be safely diagnosed and cannot be detected or ruled out with high diagnostic accuracy based on a physical examination alone [59–63]. This explains the lower grade that was assigned to the recommendation that patients with clinical signs and symptoms of a pelvic ring injury or an unstable pelvic ring injury and haemodynamic instability should be managed with a pelvic binder. It is surely advisable to use a pelvic binder if there is reason to believe that a complex pelvic ring injury is the cause of haemodynamic instability. There are no clinical studies that provide evidence suggesting that the early application of a pelvic binder can prevent haemorrhagic shock in patients with a pelvic ring injury.

An evaluation of various clinical studies on the prehospital and early clinical application of pelvic binders even suggests that these devices have only limited effectiveness or no effectiveness at all [66–69]. None of these studies presented evidence that the prehospital use of a pelvic binder reduced blood loss or mortality. It must be noted, however, that not all patients had an isolated pelvic ring fracture and that there might have been another (main) source of bleeding that was uncontrollable by a pelvic binder. Study results might have been affected by an imprecise definition of indications for use, an insufficient clinical evaluation, different levels of injury severity in the compared groups, or an inappropriate or incorrect use of pelvic binders. A pelvic binder cannot save the life of a patient with haemorrhage that is uncontrollable by mechanical pelvic ring stabilisation. Likewise, patients with pelvic ring injuries who do not bleed from the pelvis will not have an improved outcome as a result of the belt [53].

The situation is similar when it comes to concerns or contraindications regarding the use of pelvic binders. The authors find that the individual cases that are reported in the literature do not demonstrate a clear causal relationship between the application of a pelvic binder and a deterioration in the clinical course. The experts believe that there are no contraindications for the use of pelvic binders in the prehospital phase. Care, however, should be taken to ensure that these devices are used for appropriate indications and applied correctly.

For treatment to be successful, it is important to reduce the pelvic ring with mild medial rotation of the legs prior to the application of a pelvic binder, which must be correctly positioned at the level of the greater trochanters and may require careful correction of leg length discrepancy [65].

Compared with a pelvic C-clamp, which is an invasive stabilisation technique only to be used in the hospital setting, pelvic binders showed no disadvantages and were applied more rapidly. Commercial devices are superior to pelvic wrapping with a sheet [25]. Although commercial pelvic binders are widely available today, the expert group does not necessarily discourage the use of improvised devices. The included literature does not allow the recommendation of specific devices.

### Injuries to the extremities

(refers to recommendation 5)

#### Pressure dressings

(refers to recommendation 6–8)

Although there is no high-quality evidence regarding the application of manual pressure to control haemorrhage, practical experience shows that “pressure stops any bleeding” is a simple rule that is universally valid. Manual pressure using gauze or similar material is the technique of first choice.

If the pressure manoeuvre can stop the bleeding in such a way that the bleeding does not continue through the dressing, the application of pressure should not be discontinued and the dressing should not be removed since this can cause rebleeding. Surgical wound management including exploration and surgical haemorrhage control is performed in the hospital setting. For this reason, pressure must be maintained until the patient arrives at the hospital.

• If bleeding cannot be controlled by manual pressure, a pressure dressing must be applied. In order to apply pressure to the wound, a pad (e.g. a roll of gauze) is placed directly over the wound and is firmly wrapped with a bandage. All-in-one bandages, which include a dressing and an integral pressure bar, do not require a separate pad or pressure applicator. If a pressure bandage cannot control bleeding adequately, the dressing or the application of pressure on the wound must be improved. For example, a second pressure dressing can be placed over the initial dressing, or it may be more effective to completely remove the initial dressing and apply a new one. Following the application of pressure dressings, distal blood flow as well as motor and sensory function must be assessed and monitored.

• If bleeding persists, attempts must be made to stop arterial inflow proximal to the site of injury. The application of a tourniquet is recommended in the civilian setting as the final stage of escalation [27–29, 70].

#### Tourniquets

(refers to recommendations 9 and 10)

There is a paucity of studies investigating the effectiveness of tourniquets in the civilian setting. Henry et al. found that the use of tourniquets was associated with improved patient survival and decreased blood transfusion requirements and did not influence the rate of delayed amputations [29]. In a retrospective study, Taghavi et al. found that the use of tourniquets neither resulted in a survival advantage nor had any disadvantages [27].

Tourniquets can safely, rapidly and effectively control bleeding from an open extremity injury and should be used when initial measures, i.e. direct pressure and pressure dressings, are unsuccessful [71]. If bleeding can be adequately controlled with a tourniquet, the time of application should be documented and the tourniquet should be left in place until surgical management. Since tourniquet use can lead to an increase in mean arterial pressure, it must continually be monitored closely. Only then can the recurrence of bleeding be prevented.

Since tourniquet use is potentially associated with an increased risk of nerve and vascular damage, it should, whenever possible, be converted to another method of haemostasis. This applies in particular to injuries that can be adequately managed with gentler bleeding control methods, especially in situations in which the source of bleeding was not readily accessible (e.g. in trapped patients) and bleeding (in extricated patients) was found to be less severe than initially feared. The opinion of the expert group is that, whenever possible, tourniquet conversion should be considered in these cases.

Tourniquets can help reduce mortality and are associated with low rates of complications such as nerve paralysis, compartment syndrome, or secondary amputation [28, 29]. The loss of a limb from a tourniquet is rare [72]. As with other emergency techniques, the tourniquet should not be used for the first time in an emergency, but should be learned under supervision and practiced regularly. 

#### Haemostatic agents

(refers to recommendations 11–13)

Only a few comparative studies are available which address the effectiveness of haemostatic agents in severely injured patients. Currently, the evidence base is best for the effectiveness of chitosan. Kabeer et al. showed that the application of chitosan dressings significantly reduced time to haemostasis and blood loss in patients with bleeding scalp wounds when compared with standard gauze dressings [73]. Likewise, Hatamabadi et al. found that chitosan-coated gauze reduced the time to haemorrhage control and the loss of blood when compared with conventional pressure dressings [34]. Since the study of Hatamabadi et al. only included patients who had sustained a stab wound with a minimum wound length of 3 cm, the recommendation has included these 3 cm. In a registry study on patients with an ISS > 15, Winstanley et al. demonstrated an association between the use of haemostatic agents and improved survival, mostly in patients with more severe injuries. This was particularly evident in patients who had chitosan dressings [36].

There are currently no studies addressing the effectiveness of other haemostatic agents in the management of severely injured patients. Since it cannot be ruled out that these agents are effective as well, the general recommendation is that haemostatic dressings can be used as an adjunct.

The following haemostatic agents are available.

Chitin and chitosan.

Chitin and chitosan are polysaccharide biopolymers. As haemostatic agents, they induce vasoconstriction and rapidly activate red blood cells, platelets, and coagulation factors. In addition, chitosan increases platelet adhesion and aggregation at sites of damaged tissue [74]. The use of chitosan as a powder or inside a water-soluble bag is associated with disadvantages. In an animal model in which a typical projectile tract was created and the common femoral vein and artery were completely severed, chitosan was delivered to the wound using a syringe-like applicator and achieved initial haemostasis in 100% and survival in 88% of the animals [75]. Chitosan-impregnated dressings and cotton gauze (without a carrier material) are also available. High-quality evidence on the clinical use of chitosan has been reported [34, 36, 73].

Zeolite group.

Zeolites are microporous, crystalline aluminosilicates from volcanic rock. They are used as granules packaged in small bags. Their effect is based on the extremely rapid adsorption of fluid at the site of bleeding and the subsequent concentration of cellular blood components such as platelets and clotting factors. Moreover, the negative surface charge of the granules is believed to accelerate the coagulation cascade [76]. The application of zeolites leads to an exothermic reaction and high temperatures (42–140.4 °C). Similar effects have been reported for a modified zeolite [77, 78]. Burns as well as injuries to nerves and tendons have been reported to occur following the application of zeolites [79]. The evidence that is reported in studies on the efficacy of zeolites is inconclusive. There are studies that reported good results [78, 80] as well as studies that did not find zeolites to be efficacious [81–83].

Kaolin.

Kaolin is an aluminosilicate and also activates and accelerates the intrinsic coagulation pathway. A non-woven fabric is impregnated with kaolin and applied to a wound. In experimental models, these dressings showed higher survival rates and more effective bleeding control than conventional dressings [84–86].

An overall evaluation of the benefits of local haemostatic agents is, however, extremely difficult. Differences in haemorrhage models make it almost impossible to compare results for haemostatic efficacy. The most important conclusions that can be drawn from a multitude of experimental studies are the following:

1. Regardless of the haemostatic agent used, the application of pressure to the source of bleeding is essential [75]

2. Not every product is suitable for every type of bleeding [87]

Experimental studies provided sobering evidence that simple gauze dressings had similar efficacy in arresting bleeding compared to haemostatic agents [75, 88, 89]. The effectiveness may thus primarily depend on the correct placement of gauze and/or haemostatic agents (wound packing) and the application of pressure. Further studies must be conducted in order to evaluate the role of haemostatic agents in the management of severely injured patients.

### Junctional haemorrhage

In general, the recommendations for the control of bleeding from extremity injuries also apply to junctional haemorrhage (in the cervical, axillary or inguinal regions). For anatomical reasons, however, it is often difficult or even impossible to access these zones for bleeding control. Special devices (such as pneumatic compression devices) may be required to stop the bleeding. Since we did not find clinical evidence supporting the superiority of such devices, we did not give a recommendation. Further research is warranted.

### Control of femoral haemorrhage

Femur fractures are associated with increased mortality in trauma patients [90, 91]. Blood loss after femoral fractures is often underestimated, especially in patients with high-energy trauma [92].Several studies showed that trauma patients with femur fractures had increased transfusion requirements [93–95]. Since these patients can develop a clinically relevant blood loss of up to 2500 mL, femur fractures can be regarded as a cause of haemorrhagic shock [92].

Reduction and splinting of extremity injuries are usually performed to reduce pain, prevent nerve and/or vascular damage, and protect the soft tissues by relieving pressure caused by fracture fragments or the deviation of the anatomical axis. Reduction and splinting are believed to restore normal soft-tissue tension and to reduce the amount of bleeding. In a study, early immobilisation of femoral fractures with a traction splint that realigns the limb was reported to be associated with a decrease in transfusion requirements and a reduction in pulmonary complications [96]. This study was conducted on patients with isolated femoral fractures and included a high percentage of gunshot wounds. It did not meet the inclusion criteria of the present guideline. The systematic literature search that was performed on this topic did not identify any comparative studies that investigated the effect of using traction splints for haemorrhage control in patients with multiple and/or severe injuries. For this reason, we did not give a recommendation on the use of traction splints. In general, however, the reduction and immobilisation of fractures is considered appropriate. The role of reduction and splinting in controlling severe haemorrhage requires further clinical research.

### Bleeding from the head and face

#### Scalp wounds

(refers to recommendation 14)

The skin of the head is highly vasculararized tissue. Scalp wounds can therefore be associated with a considerable loss of blood and can thus contribute to haemodynamic instability [97–99].

The stepwise approach described above, i.e. manual pressure, pressure dressings, and haemostatic agents, should also be used in attempts to adequately control bleeding from the scalp. Kabeer et al. reported that the use of chitosan dressings significantly reduced time to haemostasis in patients with bleeding scalp wounds [73]. The application of such dressings thus appears to be useful and appropriate. Further options for controlling bleeding from wounds in the head and neck region are available and are presented here. The evidence, however, cannot yet be assessed conclusively.

First promising results were reported for a clamp system [98, 100, 101]. In the military setting, the use of this device has been recommended in Tactical Combat Casualty Care (TCCC) [101]. Skin stapling devices, tissue glues, etc. are further options for controlling haemorrhage [102]. On the whole, however, the evidence base for the control of bleeding in the head and neck region is poor and warrants much more research attention.

#### Epistaxis

(refers to recommendation 15)

Bleeding from wounds in the upper midfacial and nasal regions may be haemodynamically relevant if important vessels (e.g. the maxillary artery) are injured. In these cases, treatment can be imperative at the scene of injury and/or during transport. Since it can be difficult to apply direct pressure on arteries in the head and neck region (facial artery at the border of the mandible on both sides or the temporal artery at the temple), other options must be used to control bleeding in these areas in an effective and rapid manner. In emergency situations, these options include inflatable tamponades that are placed into the nose (pneumatic tamponades that are filled for example with water) or posterior and anterior nasal packing (Bellocq’s tamponade). In a clinical study, nasal packing was found to be superior to a pneumatic inflation system in terms of bleeding control, transfusion requirements, and long-term complications [37]. Nasal packing, however, requires more time and technical skills. By contrast, a pneumatic tamponade is easy to use. The study was conducted in an emergency department and not in the prehospital setting where, for example, the time, material and skills required for nasal packing are lacking. For this reason, pneumatic tamponade can be considered a useful method also in an emergency situation and should be preferred to nasal packing if the emergency response team has no experience in how to pack the nose correctly.

It should be noted that if bleeding increases after the insertion and inflation of a pneumatic tamponade (as a result of the disruption of the maxillary artery in patients with a LeFort I fracture and an increase in the fracture gap following the expansion of the pneumatic system), the tamponade must be removed, and either the maxilla must be manually compressed against the midface or nasal packing must be performed to stop bleeding in such situations.

## Limitations of the guideline

When the literature that provides the basis for the key recommendations in this chapter is assessed, attention must be drawn to a number of limitations that were taken into consideration in the interpretation of results. For this chapter dealing with the treatment of pre-hospital bleeding, internal bleeding sources (i.e. intraabdominal, intrathoracal or intracranial haemorrhage) were not addressed with a PICO question, because there currently is no direct pre-hospital therapy available for such bleedings. For ethical reasons, controlled clinical studies on severe haemorrhage and bleeding control methods are unlikely to be feasible. A major reason is the absence of equipoise, i.e. there is no equivalent medical alternative to bleeding control. A non-treatment control group is not acceptable to both patients and care providers and cannot be advocated.

There are three major aspects that can distort the results of studies:

A) Clinical heterogeneity.

Most of the studies that were identified in the literature search are retrospective in nature, and many of them analysed registry data. Interventions often did not follow a prospectively defined protocol but were performed at the discretion of the care provider. In many cases, there is no accurate description of how an intervention was implemented. For this reason, it is often unclear whether an intervention was always performed in an identical and technically correct manner. These studies are of low value, and it is no surprise that they report contradictory results.

According to the experience of the expert group and evidence from the literature, interventions were not always implemented correctly and were therefore not effective or not as effective as expected. Even trained teams often do not proficiently perform interventions in clinical practice. A study on the prehospital use of pelvic binders by trained personnel revealed that only 80% of participants placed binders correctly [103]. Studies on the application of tourniquets reported different design-dependent success rates [104–108]. An analysis of treatment failures showed that the devices were often ineffective because they had been applied too loosely before twisting [108–110].In these cases, treatment failed because of misuse.

B) Selection bias.

Most studies investigated patients who arrived alive at a hospital. Patients with injuries who would have benefitted from an intervention that they did not receive may not have reached a hospital because they bled to death at the scene or during transport. Accordingly, there is a risk that a study only included patients who had a lower risk of exsanguination than patients who died in the prehospital setting. When these studies did not detect a measurable effect, this does not mean that the intervention was ineffective but rather that the included patients did not benefit from the intervention because their injuries were not severe enough or there was no indication for the intervention.

C) Relevance and comparability.

In clinical reality, haemorrhages are heterogeneous in terms of the amount of blood loss, the type of bleeding vessel (arterial, venous, diffuse, spurting haemorrhages), or individual factors such as the influence of anticoagulant medications on the blood’s ability to coagulate.

Simply put, the amount of blood loss depends on the size of the “hole” in the vessel, hydrostatic pressure, and the blood’s ability to coagulate [111]. These parameters cannot be controlled in clinical studies. The accurate assessment of external blood loss too is a challenge for emergency physicians and emergency medical services personnel [112]. As a result, clinical studies that do not exactly define the types of haemorrhage that are addressed may investigate heterogeneous study populations with widely different injuries. At the same time, the effectiveness of blood control can considerably depend on the type and severity of haemorrhage. The application of manual pressure to a junctional vessel is less effective than the application of manual pressure to stop bleeding from a vein at a peripheral site of the body.

There are experimental models that address not only uncontrolled bleeding from a vessel but also impaired coagulation with hypovolaemia. These models, however, often do not reflect the reality of massive bleeding adequately and even to the point of clinical absurdity [111]. In addition, experimental models are associated with a low level of evidence which is not sufficient to develop a guideline at the highest level (Level S3) and with a guideline with all elements of systematic development. For this reason, they are excluded from this analysis.

Many studies on bleeding control were performed in the military setting and often cannot be compared with studies involving civilian emergency medical service systems when it comes to injury types and timelines. In particular, the differences in timelines (i.e. delayed evacuation from a combat situation) were the primary reason for excluding military research concerning tourniquet use. Furthermore, there are differences in training, medical skills, and available equipment. For example, it is difficult to transfer endpoints such as nerve damage or rates of amputation after tourniquet use in the military setting to the civilian system since the duration of tourniquet application (evacuation from the combat zone alone can be substantially longer than the entire period of prehospital care in the civilian setting) and the radicality of tourniquet application (as proximal as possible in the military setting and as distal as possible in the civilian system) are different. For this reason, the experts decided not to include evidence from the military setting in the formulation of key recommendations on the use of tourniquets.

Patient values and preferences were sought but not received. The effect of this on the guideline is unclear, and there is a lack of research evidence on the effect of patient participation on treatment decisions or outcomes in the emergency setting.

## Unanswered questions and future research

There are no comparative studies addressing the role of controlling active bleeding. For this reason, a GPP (strong recommendation 1) was formulated. Comparative studies that meet the requirements of an S3-level guideline are unfeasible and unlikely to be ethically acceptable since there are no alternative options to haemorrhage control and uncontrolled bleeding can lead to death from exsanguination. This means that only different bleeding control methods can be compared.

Moreover, there are no studies assessing the role of rapid patient transport to a hospital for surgical haemorrhage control. Although there is empirical evidence supporting this approach, data suggesting that rapid patient transportation is associated with benefits such as improved survival are hardly convincing (no recommendation).

The role of an assessment of mechanical stability of the pelvis is unclear. There is evidence suggesting that this examination provides key information regarding the necessity of external stabilisation. The literature does not provide data revealing that mechanical stability testing can have adverse effects and in particular can lead to rebleeding or poorer treatment outcomes. It is unclear whether the early application of a pelvic binder can (prophylactically) prevent haemorrhagic shock. Likewise, it is unclear whether there are situations in which a pelvic binder may have adverse effects and which therefore should be considered contraindications (GPP, strong recommendation 2; recommendation 3).

There is empirical evidence supporting the application of manual pressure to control haemorrhage. Clinical studies confirming this are not available (strong recommendation 5). It was recommended not to perform repeated assessments of whether bleeding has stopped in the prehospital setting. Studies investigating this issue and possible negative consequences for patients (GPP, recommendation 6) are not available.

The use of pressure dressings in the management of bleeding after blunt trauma to the torso and/or the extremities has no evidence base that is comparable to that available for the use of pressure dressings in patients with bleeding from penetrating torso and/or extremity injuries. There is, however, no obvious reason why this approach should not be used for both mechanisms of injury. If higher quality studies were available, they would strengthen this recommendation for the management of blunt trauma (GPP, strong recommendation 8).

The available literature did not allow key recommendations to be formulated on the control of junctional haemorrhage and bleeding from femoral shaft fractures. A variety of bleeding control devices are commercially available. Studies that investigate the effectiveness of such devices and meet the inclusion criteria of an S3-level guideline are not available (no recommendations).

Since tourniquet use is associated with possible risks and side effects from warm ischaemia and high pressure on nerves and vessels, the duration of tourniquet use should be as short as possible. Conversion to another technique in the prehospital phase must be critically assessed. There are no studies demonstrating that conversion to another technique is possible and safe before arrival at a hospital and that it does not adversely affect treatment outcome. Such studies would have to define in detail the type of injury and the severity of haemorrhage that are investigated in order to provide guidance for treatment (GPP, recommendation 10).

Various haemostatic agents were investigated in studies that were excluded in this guideline for methodological reasons. There is no high-quality evidence demonstrating the effectiveness of such agents in the management of severely injured patients. The special influence of anticoagulant medications appears to be a particularly important issue that currently cannot be resolved (open recommendation 13). Likewise, the role of pressure using non-impregnated material is still unclear. It is possible that haemostasis is not achieved by the haemostatic agent alone but also by the pressure that is applied to bleeding vessels by the carrier material. As a consequence, non-impregnated carrier material should be used as a control group in clinical studies.

There is virtually no study that investigated the needs and requirements of patients. Patient-reported outcome measures (PROMs) such as patient comfort and pain reduction (e.g. pain associated with tourniquet use or pain reduction after fracture splinting) are certainly important aspects of many techniques addressed here.

## Conclusion

Early bleeding control in the prehospital phase of care is of central importance in the management of patients with multiple and/or severe injuries. Exsanguination is not only a major cause of preventable deaths, but a massive blood loss also has direct effects on blood coagulation and organ function. This means that massive haemorrhage has serious consequences in terms of comorbidities and increases the pressure on care providers to act.

Bleeding control is the basic objective of treatment. This can be easily justified on the basis of empirical evidence. There is, however, a lack of reliable and high-quality studies that assess and compare methods for controlling bleeding in patients with multiple and/or severe injuries.

The guideline provides reasonable and practical recommendations (although mostly with a low grade of recommendation) and also reveals a number of open research questions that can hopefully be answered when the guideline will be revised again.

## Supplementary Information

Below is the link to the electronic supplementary material.

Supplementary file1 (PDF 1388 KB)

## Data Availability

No datasets were generated or analysed during the current study.

## Abbreviations

Adj: Adjusted
AHRQ: Agency for healthcare research and quality
AIS: Abbreviated injury scale
AWMF: German association of the scientific medical societies
CG: Control group
CI: Confidence interval
d: Days
ED: Emergency department
GCS: Glasgow coma scale
GPP: Good (clinical) practice point
h: Hours
IG: Intervention group
ISS: Injury severity score
LoE: Level of evidence
MD: Mean difference
n.r.: Not reported
n.s.: Not significant
OR: Odds ratio
PCCD: Pelvic circumferential compression device
PH: Prehospital
PICO: Population, intervention, comparison, and outcome
PRISMA: Preferred reporting items for systematic reviews and meta-analyses
RCT: Randomised controlled trial
RoB: Risk of bias
SBP: Systolic blood pressure
SD: Standard deviation
SMR: Standardised mortality rate (observed divided by expected mortality, using the Revised Injury Severity Classification Score II (RISC-II) to estimate probability of survival)
Unadj: Unadjusted
VAS: Visual analogue scale
y: Years
