# Supplementary material for: Stop the bleed “ – Prehospital bleeding control in patients with multiple and/or severe injuries – A systematic review and clinical practice guideline – A systematic review and clinical practice guideline
Source: Eur J Trauma Emerg Surg. 2025 Feb 5;51(1):92. doi: 10.1007/s00068-024-02726-1 (PMC11799122; doi:10.1007/s00068-024-02726-1)
Supplement: Supplementary file 1 — Supplementary file1 (PDF 1388 KB) [file 68_2024_2726_MOESM1_ESM.pdf]

“Stop the bleed“ – Prehospital bleeding control in patients with multiple and/or severe injuries – A systematic review and clinical practice guideline

Table S1. PICO questions[1]

The target population of the guideline consists of adults (≥14 years) with suspected polytrauma or trauma-related severe injury (ISS ≥16 and/or GCS <9) in the prehospital phase of care.

	Population	Intervention(s)	Control(s)	Outcome(s)
1	target population, injury to the trunk/pelvis, with active bleeding	prehospital REBOA, thoracotomy and manual compression of the aorta	unspecified	bleeding control, mortality (especially early: 6h, 24h), transfusion requirements, haemodynamic stability, other clinically relevant outcomes
2	target population, pelvic injury, with active bleeding	prehospital pelvic belt, pelvic sheeting, other emergency mechanical stabilisation	unspecified	bleeding control, mortality (especially early: 6h, 24h), transfusion requirements, haemodynamic stability, other clinically relevant outcomes
3	target population, injury in transitional regions (junctional/body trunk proximal), with active bleeding	prehospital manual compression, junctional tourniquet	not specified	bleeding control, prehospital survival, transfusion requirements, haemodynamic stability on reaching the hospital, mortality (early/24 h, in-hospital mortality), length of ICU stay
4	target population, extremity injury, with active bleeding	prehospital manual compression, compression bandages, compression devices, wound packing, haemostatic agents, tourniquet	not specified	bleeding control, prehospital survival, transfusion requirements, haemodynamic stability on reaching the hospital, mortality (early/24 h, in-hospital mortality), length of ICU stay
5	target population, limb injury, with active bleeding<br>alternatively, specific subpopulations (to allow defining indications for the intervention)	prehospital tourniquet	no tourniquet	bleeding control, prehospital survival, transfusion requirements, haemodynamic stability on reaching the hospital, mortality (early/24 h, in-hospital mortality), length of ICU stay

[1] Table translated from German using DeepL Translate (https://www.deepl.com/translator) with manual modifications

	Population	Intervention(s)	Control(s)	Outcome(s)
6	target population, with femoral shaft fracture	prehospital traction splints (e.g. Thomas splint)	not specified	bleeding control, prehospital survival, transfusion requirements, haemodynamic stability on reaching the hospital, mortality (early/24 h, in-hospital mortality), length of ICU stay
7	target population, with life-threatening haemorrhage on anticoagulation (specifically: thrombin inhibitors)	haemostatic agents (specifically: chitosan)	compression by manual pressure or pressure bandage	bleeding control, other clinically relevant outcomes
8	target population, with head/facial injuries and active bleeding or epistaxis	manual compression, pressure dressing, tamponades, balloon catheters (Bellocq tamponade), wound clamps, tamponades made of hard fat with bismuth and tannin (e.g. Stryphnasal® N nasal stick), topical tranexamic acid	not specified	bleeding control, prehospital survival, transfusion requirements, haemodynamic stability on reaching the hospital, mortality (early/24 h, in-hospital mortality), length of ICU stay

Table S2. Literature search details

Search strategy 2021, MEDLINE (via Ovid)	Date: 23.06.2021	650 Hits

1. exp Multiple Trauma/
2. (polytrauma* or trauma patient? or (severe adj2 shock)).ti,ab,kf.
3. ((multiple or major or severe* or serious*) adj3 (trauma* or injur*)).ti,ab,kf.
4. ((blunt or penetrating) adj5 (trauma* or injur*)).ti,ab,kf.
5. (*Critical Care/ or *Emergencies/ or (life threatening or critical care or emergen*).ti,ab,kf.) and (trauma* or injur*).ti,ab,kf.
6. 1 or 2 or 3 or 4 or 5
7. exp animals/ not humans.sh.
8. (comment or editorial or letter).pt. or case report*.mp.
9. (pelvic adj1 (binder? or binding or sheet? or sheeting)).ti,ab,kf.
10. circumferential compression.ti,ab,kf.
11. (((pressure or compression) adj2 (bandage? or dressing?)) or tourniquet? or ((wound or pelvic or preperitoneal or extraperitoneal) and packing)).ti,ab,kf. or exp Tourniquets/ or *Compression Bandages/ or (traction splint? or thomas splint?).ti,ab,kf.
12. (exp Chitosan/ or (h?emostyptic or chitosan or Celox or QuikClot or HemCon).ti,ab,kf.) and (exp Hemorrhage/ or exp Exsanguination/ or (bleed* or h?emorrhag* or exsanguination).ti,ab,kf.)
13. ((nasopharyngeal or nasal or bellocq) adj2 (tamponade or packing or balloon)).ti,ab,kf.
14. (massive or life-threatening).ti,ab,kf. and (exp Epistaxis/ or epistaxis.ti,ab,kf.)
15. (wound clamp or itclamp).ti,ab,kf.
16. (injectable adj3 sponge?).ti,ab,kf.
17. 9 or 10 or 11 or 12 or 13 or 15 or 16
18. 6 and 17
19. 18 not 7
20. 19 not 8

Search strategy 2021, Embase (via Elsevier)	Date: 23.06.2021	147 Hits

#1 'multiple trauma'/exp

#2 (polytrauma* OR "trauma patient?"):ti,ab,kw OR (severe NEXT/2 shock):ti,ab,kw

#3 ((multiple OR major OR severe* OR serious*) NEXT/3 (trauma* OR injur*)):ti,ab,kw

#4 ((blunt OR penetrating) NEXT/5 (trauma* OR injur*)):ti,ab,kw

#5 ('intensive care'/mj OR 'emergency'/mj OR ("life threatening" OR "critical care" OR emergen*):ti,ab,kw) AND (trauma* OR injur*):ti,ab,kw

#6 #1 OR #2 OR #3 OR #4 OR #5

#7 'animals'/exp NOT 'humans'/de

#8 (comment OR editorial OR letter):it OR "case report*":ti,ab,kw

#9 [embase]/lim

#10 embase NOT (embase AND medline)

#11 (pelvic NEAR/1 (binder? OR binding OR sheet? OR sheeting)):ti,ab,kw OR 'pelvic binder'/exp

#12 "circumferential compression":ti,ab,kw

#13 (((pressure OR compression) NEAR/2 (bandage? OR dressing?)) OR tourniquet? OR ((wound OR pelvic OR preperitoneal OR extraperitoneal) AND packing)):ti,ab,kw OR 'tourniquet'/exp OR 'compression bandage'/exp OR ("traction splint?" OR "thomas splint?"):ti,ab,kw OR 'traction splint'/exp OR 'Thomas splint'/exp

#14 ('chitosan'/exp OR (h?emostyptic OR chitosan OR Celox OR QuikClot OR HemCon):ti,ab,kw) AND ('bleeding'/exp OR 'exsanguination'/exp OR (bleed* OR h?emorrhag* OR exsanguination):ti,ab,kw)

#15 ((nasopharyngeal OR nasal OR bellocq) NEAR/2 (tamponade OR packing OR balloon)):ti,ab,kw OR 'epistaxis balloon'/exp

#16 (massive OR life-threatening):ti,ab,kw AND ('epistaxis'/exp OR epistaxis:ti,ab,kw)

#17 ("wound clamp" OR itclamp):ti,ab,kw

#18 (injectable NEAR/3 sponge?):ti,ab,kw

#19 #11 OR #12 OR #13 OR #14 OR #15 OR #16 OR #17 OR #18

#20 #6 AND #17

#21 #20 NOT #7

#22 #21 NOT #8

#23 #22 AND #9

#24 #23 AND #10

#24 AND ('article'/it OR 'article in press'/it OR 'review'/it)

Table S3. List of excluded studies

Authors	Year	Title	Reason for exclusion
M. Alonso-Algarabel, X. Esteban-Sebastia, A. Santillan-Garcia, Vila-C and R. el	2019	Tourniquet use in out-of-hospital emergency care: a systematic review	Language
A. C. Beekley, J. A. Sebesta, L. H. Blackbourne, G. S. Herbert, D. S. Kauvar, D. G. Baer, T. J. Walters, P. S. Mullenix, J. B. Holcomb and G. st Combat Support Hospital Research	2008	Prehospital tourniquet use in Operation Iraqi Freedom: effect on hemorrhage control and outcomes	Study type/Outcome
C. Y. BenItez, P. Ottolino, B. M. Pereira, D. S. Lima, A. Guemes, M. Khan and M. A. F. Ribeiro Junior	2021	Tourniquet use for civilian extremity hemorrhage: systematic review of the literature	Study type/Outcome
A. Benov, I. Shkolnik, E. Glassberg, R. Nadler, S. Gendler, B. Antebi, J. Chen, N. Fink and T. Bader	2019	Prehospital trauma experience of the Israel defense forces on the Syrian border 2013-2017	Study type/Outcome
S. Brodie, T. J. Hodgetts, J. Ollerton, J. McLeod, P. Lambert and P. Mahoney	2007	Tourniquet use in combat trauma: UK military experience	Study type/Outcome
S. Brodie, T. J. Hodgetts, J. Ollerton, J. McLeod, P. Lambert and P. Mahoney	2009	Tourniquet use in combat trauma: U.K. military experience	Study type/Outcome
M. A. Brown, M. R. Daya and J. A. Worley	2009	Experience with chitosan dressings in a civilian EMS system	Study type/Outcome
N. P. Charlton, J. M. Swain, J. L. Brozek, M. Ludwikowska, E. Singletary, D. Zideman, J. Epstein, A. Darzi, A. Bak, S. Karam, Z. Les, J. N. Carlson, E. Lang and R. Nieuwlaat	2021	Control of Severe, Life-Threatening External Bleeding in the Out-of-Hospital Setting: A Systematic Review	Study type/Outcome
O. Chiara, S. Cimbanassi, G. Bellanova, M. Chiarugi, A. Mingoli, G. Olivero, S. Ribaldi, G. Tugnoli, S. Basilico, F. Bindi, L. Briani, F. Renzi, P. Chirletti, G. Di Grezia, A. Martino, R. Marzaioli, G. Noschese, N. Portolani, P. Ruscelli, M. Zago, S. Sgardello, F. Stagnitti and S. Miniello	2018	A systematic review on the use of topical hemostats in trauma and emergency surgery	Population
M. P. Cornelissen, Br, A. wijk, L. Schoonmade, G. Giannakopoulos, S. van Oostendorp and L. Geeraedts, Jr.	2020	The safety and efficacy of improvised tourniquets in life-threatening hemorrhage: a systematic review	Study type/Outcome
E. D. Cox, M. A. Schreiber, J. McManus, C. E. Wade and J. B. Holcomb	2009	New hemostatic agents in the combat setting	Study type/Outcome
M. J. El Sayed, H. Tamim, A. Mailhac and N. C. Mann	2017	Trends and Predictors of Limb Tourniquet Use by Civilian Emergency Medical Services in the United States	Population
J. Granville-Chapman, N. Jacobs and M. J. Midwinter	2011	Pre-hospital haemostatic dressings: a systematic review	Study type/Outcome

Authors	Year	Title	Reason for exclusion
A. Hoch, S. Zeidler, P. Pieroh, C. Josten, F. M. Stuby, S. C. Herath and R. German Pelvic Trauma	2021	Trends and efficacy of external emergency stabilization of pelvic ring fractures: results from the German Pelvic Trauma Registry	Intervention
B. Hossfeld, R. Lechner, F. Josse, M. Bernhard, F. Walcher, M. Helm and M. Kulla	2018	[Prehospital application of tourniquets for life-threatening extremity hemorrhage : Systematic review of literature]	Study type/Outcome
J. Kalish, P. Burke, J. Feldman, S. Agarwal, A. Glantz, P. Moyer, R. Serino and E. Hirsch	2008	The return of tourniquets. Original research evaluates the effectiveness of prehospital tourniquets for civilian penetrating extremity injuries	Study type/Outcome
S. Khanna and A. B. Dagum	2012	A critical review of the literature and an evidence-based approach for life-threatening hemorrhage in maxillofacial surgery	Study type/Outcome
H. Khoshmohabat, S. Paydar, A. Makarem, M. Y. Karami, N. Dastgheib, S. A. H. Zahraei, R. Rezaei and G. S. Mahmoudi Nezhad	2019	A review of the application of cellulose hemostatic agent on trauma injuries	Study type/Outcome
J. F. Kragh, Jr., M. L. Littrel, J. A. Jones, T. J. Walters, D. G. Baer, C. E. Wade and J. B. Holcomb	2011	Battle casualty survival with emergency tourniquet use to stop limb bleeding	Study type/Outcome
J. F. Kragh, O. N. ML, D. F. Beebe, C. J. Fox, A. C. Beekley, J. S. Cain, D. L. Parsons, R. L. Mabry and L. H. Blackbourne	2011	Survey of the indications for use of emergency tourniquets	Study type/Outcome
J. F. Kragh, Jr., T. J. Walters, D. G. Baer, C. J. Fox, C. E. Wade, J. Salinas and J. B. Holcomb	2009	Survival with emergency tourniquet use to stop bleeding in major limb trauma	Population
R. C. Kue, E. S. Temin, S. G. Weiner, J. Gates, M. H. Coleman, J. Fisher and S. Dyer	2015	Tourniquet Use in a Civilian Emergency Medical Services Setting: A Descriptive Analysis of the Boston EMS Experience	Study type/Outcome
D. Lakstein, A. Blumenfeld, T. Sokolov, G. Lin, R. Bssorai, M. Lynn and R. Ben-Abraham	2003	Tourniquets for hemorrhage control on the battlefield: a 4-year accumulated experience	Study type/Outcome
J. Leonard, J. Zietlow, D. Morris, K. Berns, S. Eyer, K. Martinson, D. Jenkins and S. Zietlow	2016	A multi-institutional study of hemostatic gauze and tourniquets in rural civilian trauma	Population
G. Ode, J. Studnek, R. Seymour, M. J. Bosse and J. R. Hsu	2015	Emergency tourniquets for civilians: Can military lessons in extremity hemorrhage be translated?	Population
A. N. Pollak, F. Battistella, J. Pettey, S. A. Olson and M. W. Chapman	1997	Reamed femoral nailing in patients with multiple injuries. Adverse effects of tourniquet use	Study type/Outcome
M. H. Scerbo, J. B. Holcomb, E. Taub, K. Gates, J. D. Love, C. E. Wade and B. A. Cotton	2017	The trauma center is too late: Major limb trauma without a pre-hospital tourniquet has increased death from hemorrhagic shock	Population
S. G. Schauer, M. D. April, J. F. Naylor, J. K. Maddry, A. A. Arana, M. A. Dubick, A. D. Fisher, C. W. Cunningham and A. E. Pusateri	2018	Prehospital Application of Hemostatic Agents in Iraq and Afghanistan	Population
P. Schober, G. Giannakopoulos, S. A. Loer and L. A. Schwarte	2019	Hemorrhage Treatment Adjuncts in a Helicopter Emergency Medical Service	Study type/Outcome

Authors	Year	Title	Reason for exclusion
G. Shao, M. Zhao, S. Yang, F. Chen, J. Chen, G. Yang and D. Yuan	2017	A new double-lumen hemostatic device for treatment of intractable traumatic epistaxis induced by craniofacial basicranial fractures	Population
J. P. Stannard, J. T. Robinson, E. R. Anderson, G. McGwin, Jr., D. A. Volgas and J. E. Alonso	2006	Negative pressure wound therapy to treat hematomas and surgical incisions following high-energy trauma	Population
O. Thabouillot, K. Bertho, E. Rozenberg, N. C. Roche, G. Boddaert, D. Jost and J. P. Tourtier	2018	How many patients could benefit from REBOA in prehospital care? A retrospective study of patients rescued by the doctors of the Paris fire brigade	Intervention
J. P. Tourtier, B. Palmier, K. Tazarourte, M. Raux, E. Meaudre, S. Ausset, A. Sailliol, B. Vivien, L. Domanski and P. Carli	2013	The concept of damage control: extending the paradigm in the prehospital setting	Study type/Outcome
T. Upile, W. Jerjes, F. Sipaul, M. El Maaytah, S. A. Nouraei, S. Singh, C. Hopper and A. Wright	2007	The role of surgical audit in improving patient management	Study type/Outcome
E. A. P. van Leent, B. van Wageningen, O. Sir, E. Hermans and J. Biert	2019	Clinical Examination of the Pelvic Ring in the Prehospital Phase	Study type/Outcome
S. E. van Oostendorp, E. C. Tan and L. M. Geeraedts, Jr.	2016	Prehospital control of life-threatening truncal and junctional haemorrhage is the ultimate challenge in optimizing trauma care	Study type/Outcome
D. O. Verbeek, M. Sugrue, Z. Balogh, D. Cass, I. Civil, I. Harris, T. Kossmann, S. Leibman, V. Malka, A. Pohl, S. Rao, M. Richardson, M. Schuetz, C. Ursic and V. Wills	2008	Acute management of hemodynamically unstable pelvic trauma patients: time for a change? Multicenter review of recent practice	Study type/Outcome
N. M. Walker, W. Eardley and J. C. Clasper	2014	UK combat-related pelvic junctional vascular injuries 2008-2011: implications for future intervention	Study type/Outcome
N. Wongtongkam	2021	Systematic review: Do commercial tourniquets have potential to be a life-saving intervention for ambulance services?	Study type/Outcome
G. Z. Xu, W. Li, K. G. Liu, W. Wu, W. C. Lu, J. F. Zhang and M. D. Wang	2014	Early pressure dressing for the prevention of subdural effusion secondary to decompressive craniectomy in patients with severe traumatic brain injury	Population
H. Bedri, H. Ayoub, J. M. Engelbart, M. Lilienthal, C. Galet and D. A. Skeete	2021	Tourniquet Application for Bleeding Control in a Rural Trauma System: Outcomes and Implications for Prehospital Providers	Study type/Outcome
L. Froberg, Helgstr, F. , C. Clausen, J. Steinmetz and H. Eckardt	2016	Mortality in trauma patients with active arterial bleeding managed by embolization or surgical packing: An observational cohort study of 66 patients	Intervention
M. Harfouche, K. Inaba, J. Cannon, M. Seamon, E. Moore, T. Scalea and J. DuBose	2021	Patterns and outcomes of zone 3 REBOA use in the management of severe pelvic fractures: Results from the AAST Aortic Occlusion for Resuscitation in Trauma and Acute Care Surgery database	Intervention
S. Magnone, F. Coccolini, R. Manfredi, D. Piazzalunga, R. Agazzi, C. Arici, M. Barozzi, G. Bellanova, A. Belluati, G. Berlot, W. Biffl, S. Camagni, L. Campanati, C. C. Castelli, F. Catena, O. Chiara, N.	2014	Management of hemodynamically unstable pelvic trauma: results of the first Italian consensus conference (cooperative guidelines of the Italian Society of Surgery, the Italian Association of Hospital Surgeons, the Multi-specialist Italian Society of Young Surgeons, the Italian Society of Emergency Surgery and	Intervention

Authors	Year	Title	Reason for exclusion
Colaianni, S. De Masi, S. Di Saverio, G. Dodi, A. Fabbri, G. Faustinelli, G. Gambale, M. G. Capponi, M. Lotti, G. Marchesi, A. Masse, T. Mastropietro, G. Nardi, R. Niola, G. E. Nita, M. Pisano, E. Poiasina, E. Poletti, A. Rampoldi, S. Ribaldi, G. Rispoli, L. Rizzi, V. Sonzogni, G. Tugnoli and L. Ansaloni		Trauma, the Italian Society of Anesthesia, Analgesia, Resuscitation and Intensive Care, the Italian Society of Orthopaedics and Traumatology, the Italian Society of Emergency Medicine, the Italian Society of Medical Radiology -Section of Vascular and Interventional Radiology- and the World Society of Emergency Surgery)	
P. Roman, A. Rodriguez-Alvarez, D. Bertini-Perez, C. Ropero-Padilla, L. Martin-Ibanez and M. Rodriguez-Arrastia	2021	Tourniquets as a haemorrhage control measure in military and civilian care settings: An integrative review	Study type/Outcome
R. Rossaint, B. Bouillon, V. Cerny, T. J. Coats, J. Duranteau, Fern, E. ez-Mondejar, B. J. Hunt, R. Komadina, G. Nardi, E. Neugebauer, Y. Ozier, L. Riddez, A. Schultz, P. F. Stahel, J. L. Vincent, D. R. Spahn and T. Task Force for Advanced Bleeding Care in	2010	Management of bleeding following major trauma: an updated European guideline	Study type/Outcome
S. G. Schauer, M. D. April, J. F. Naylor, J. Wiese, K. L. Ryan, A. D. Fisher, C. W. Cunningham, N. Mitchell and M. A. Antonacci	2017	Prehospital Administration of Tranexamic Acid by Ground Forces in Afghanistan: The Prehospital Trauma Registry Experience	Intervention
S. Smith, J. White, K. N. Wanis, A. Beckett, V. C. McAlister and R. Hilsden	2019	The effectiveness of junctional tourniquets: A systematic review and meta-analysis	Population
D. R. Spahn, V. Cerny, T. J. Coats, J. Duranteau, Fern, E. ez-Mondejar, G. Gordini, P. F. Stahel, B. J. Hunt, R. Komadina, E. Neugebauer, Y. Ozier, L. Riddez, A. Schultz, J. L. Vincent, R. Rossaint and T. Task Force for Advanced Bleeding Care in	2007	Management of bleeding following major trauma: a European guideline	Study type/Outcome
P. Schober, G. Giannakopoulos, S. A. Loer and L. A. Schwarte	2019	Hemorrhage Treatment Adjuncts in a Helicopter Emergency Medical Service	Study type/Outcome
M. Y. Shah Jahan, M. A. Shamila, N. Nurul Azlean, M. Mohd Amin, An, K. akumar, K. B. Ahmad Ibrahim, M. N. Ahmad Tajuddin, T. Aik Howe, M. Md Saed, M. Fatahul Laham, M. I. Ridzuan, Z. Mohd Idzwan, M. Y. Mohd Khairizam, J. Mathew, M. Fitzgerald, J. Sabariah Faizah and G. Kiat Kee	2019	Administration of tranexamic acid for victims of severe trauma within pre-hospital care ambulance services (PHCAS) in Malaysia	Intervention
S. G. Cornero, M. Maegele, R. Lefering, C. Abbati, S. Gupta, F. Sammartano, S. Cimbanassi and O. Chiara	2020	Predictive Factors for Massive Transfusion in Trauma: A Novel Clinical Score from an Italian Trauma Center and German Trauma Registry	Intervention
S. Halewyck, C. Depuydt and O. Michel	2016	Severe traumatic epistaxis	Study type/Outcome

Table S4. Evidence Tables

Pelvic binders

Study: Reference, aim, design, setting	Participants: selection criteria, characteristics	N Participants; Intervention (IG) vs. Control group (CG)	Main outcomes	Assessment: LoE, risk of bias; Conclusions
**Berger-Groch (2021)**<br>"Evaluation of pelvic circular compression devices in severely injured trauma patients with pelvic fractures" DOI: 10.1080/10903127.2021.1945717<br>**Study design**<br>Comparative registry study<br>(TraumaRegister DGU®)<br>**Aim of the study**<br>"The purpose of the current investigation is to determine whether patients with significant pelvic trauma, treated with a PCCD, have decreased mortality and a lower risk for blood loss."<br>**Setting**<br>Germany 2015-2016	**Inclusion criteria**<br>• patients suffering from a relevant pelvic trauma (AIS severity 3-5; unstable fractures with or without relevant blood loss or open fracture)<br>• ISS ≥9<br>• age ≥16 years<br>• directly admitted from the scene of the accident to the participating hospital<br>• complete outcome documentation (survival to hospital discharge or death)<br>**Exclusion criteria**<br>• patients transferred from another hospital (no prehospital data available)<br>• patients transferred to another hospital within 48 h (outcome unknown)<br>• no information about the use of PCCD, either prehospital or in the ED<br>**Characteristics**<br>Age [y], mean ± SD<br>no PCCD: 51.3 ± 20.6<br>PH-PCCD:46.9 ± 19.3<br>ED-PCCD: 53.0 ± 19.0, p=0.001<br>Male, n (%)<br>no PCCD: 385 (59.3)<br>PH-PCCD: 181 (63.7)<br>ED-PCCD: 99 (58.2), p=0.37	**Participants**<br>N=1103 patients<br>**Study groups**<br>no PCCD: patients without PCCD stabilization (N=649)<br>PH-PCCD: patients receiving PCCD stabilization in the prehospital phase (N=284)<br>ED-PCCD: patients receiving PCCD stabilization in the resuscitation phase in the emergency department (N=170)<br>No information on the type of PCCD used, no confirmation that it had been properly fitted.<br>Missing data for pelvic binder were not replaced; 11% of patients had missing data for PH PCCD, and 8% of cases had missing data for ED PCCD.<br>**Adjusting variables in multivariable logistic regression**<br>• age (8 categories)<br>• sex<br>• prehospital shock<br>• shock on admission<br>• cardio-pulmonary resuscitation (CPR)<br>• unconsciousness (GCS≤8)<br>• prehospital intubation<br>• prehospital catecholamines<br>• ISS	**Adjusted outcomes**<br>In-hospital mortality, adjusted OR (95% CI)<br>no PCCD: reference<br>PH-PCCD: 1.493 (0.802-2.780), p=0.206<br>ED-PCCD: 1.453 (0.709-2.974), p=0.307<br>In-hospital mortality, O/E ratio (95% CI)[§]<br>no PCCD: 0.910 (0.721-1.100)<br>PH-PCCD: 1.033 (0.815-1.251)<br>ED-PCCD: 1.161 (0.875-1.448)<br>Transfusion, adjusted OR (95% CI)<br>no PCCD: reference<br>PH-PCCD: 1.607 (1.049-2.464), p=0.029<br>ED-PCCD: 1.423 (0.847-2.389), p=0.182<br>**Unadjusted outcomes**<br>In-hospital mortality, n/N (%)<br>no PCCD: 78/649 (12)<br>PH-PCCD: 66/284 (23.2)<br>ED-PCCD: 46/170 (27.1), p<0.001<br>24h mortality, n/N (%)<br>no PCCD: 34/649 (5.2)<br>PH-PCCD: 37/284 (13)<br>ED-PCCD: 30/170 (17.6), p<0.001<br>ICU stay [d], median / mean SD<br>no PCCD: 4.0 / 9.3 ± 12.5<br>PH-PCCD: 5.0 / 11.1 ± 15.0<br>ED-PCCD: 6.0 / 12.4 ± 16.0, p=0.064	**Level of evidence**<br>2b<br>**Risk of bias**<br>Selection bias: –<br>Performance bias: ?<br>Attrition bias: +<br>Detection bias: +<br>**Authors' conclusion**<br>"Even after subsequent adjustment in this study, the postulated beneficial effect of PCCDs in terms of decreased mortality and lower needs for blood transfusion could not be confirmed. Application of PCCDs in patients with a severe pelvic trauma is a general indicator for a critical patient with increased mortality."<br>**Reviewers' conclusion**<br>The study conclusions account for the retrospective study design and substantial risk of selection bias. The groups were not balanced at

Study: Reference, aim, design, setting	Participants: selection criteria, characteristics	N Participants; Intervention (IG) vs. Control group (CG)	Main outcomes	Assessment: LoE, risk of bias; Conclusions
	ISS, mean ± SD<br>no PCCD: 27.9 ± 13.8<br>PH-PCCD: 34.12 ± 16.4<br>ED-PCCD: 35.9 ± 5.5, p<0.001<br><br>GCS, median / mean ± SD<br>no PCCD: 15 / 12.6 ± 4.1<br>PH-PCCD: 14 / 11.3 ± 4.7<br>ED-PCCD: 14 / 11.6 ± 4.4, p<0.001<br><br>Pelvic Injury Severity, p<0.001:<br>AIS_{pelvis} = 3, n (%)<br>no PCCD: 332 (51.2)<br>PH-PCCD: 94 (33.2)<br>ED-PCCD: 44 (25.9)<br><br>AIS_{pelvis} = 4, n (%)<br>no PCCD: 244 (37.6)<br>PH-PCCD: 121 (42.6)<br>ED-PCCD: 67 (39.4)<br><br>AIS_{pelvis} = 5, n (%)<br>no PCCD: 73 (11.2)<br>PH-PCCD: 69 (24.3)<br>ED-PCCD: 59 (34.7)<br><br>AIS_{Head} ≥3<br>no PCCD: 160 (24.7)<br>PH-PCCD: 68 (23.9)<br>ED-PCCD: 45 (26.5)<br><br>AIS_{Thorax}≥3<br>no PCCD: 307 (47.3)<br>PH-PCCD: 168 (59.2)<br>ED-PCCD: 103 (60.6)<br><br>$AIS_{Abdomen}$≥3	• severe pelvic trauma (AIS 4-5)<br>• relevant injuries (AIS 3+) to the head, the thorax, and the abdomen	Hospital stay [d], median / mean SD<br>no PCCD: 20.0 / 24.01 ± 18.3<br>PH-PCCD: 21.5 / 25.7 ± 24.8<br>ED-PCCD: 20.5 / 24.6 ± 24.1, p=0.78<br><br>§ observed/expected ratio; expected mortality calculated using the RISC prognosis in %	baseline, but the analysis was adjusted for important confounders.

Study: Reference, aim, design, setting	Participants: selection criteria, characteristics	N Participants; Intervention (IG) vs. Control group (CG)	Main outcomes	Assessment: LoE, risk of bias; Conclusions
	no PCCD: 102 (15.7)<br>PH-PCCD: 77 (27.1)<br>ED-PCCD: 43 (25.3)<br><br><u>Shock (SBP ≤90 prehospital), n (%)</u><br>no PCCD: 70 (11.9)<br>PH-PCCD: 69 (27.2)<br>ED-PCCD: 31 (21.7), p<0.001			
**Pierrie (2021)**<br>"Pilot randomized trial of pre-hospital advanced therapies for the control of hemorrhage (PATCH) using pelvic binders". *Am J Emergency Med* 2021; 42: 43-48.<br><br>**Study design**<br>Randomised controlled trial<br>(PATCH trial)<br><br>**Aim of the study**<br>"To determine if prehospital placement of pelvic binders is feasible (even among patients with diagnoses other than pelvic fracture) and to pilot the feasibility of conducting a randomized trial evaluating the efficacy of pelvic binder	**Inclusion criteria**<br>• ≥18 years of age<br>• high-energy traumatic injury other than a ground-level fall<br>• either had clinical signs and symptoms of pelvic ring injury (PRI, such as pelvic, hip, or groin pain, deformity, ecchymosis, or crepitus) or were hypotensive (SBP <90 mmHg)<br><br>**Exclusion criteria**<br>• ground-level fall<br>• penetrating pelvic injury without obvious evidence of fracture<br>• obviously pregnant<br>• body habitus larger than could be accommodated by a commercial pelvic binder<br>• going to be transferred to a facility other than the level-one trauma center conducting this study<br><br>**Characteristics**<br>98% blast and gunshot wounds<br><br><u>Age [y], median</u><br>IG: 33.5 vs. CG: 36.0, p=0.97	**Participants**<br>N=50 patients enrolled, N=43 consented to participate in the outcomes analysis<br><br>**Study groups**<br>IG: pelvic binder (N=20 in outcomes analysis)<br>CG: current standard of care (N=23 in outcomes analysis)<br><br>SAM Pelvic Sling II (SAM Medical Products, Wilsonville, OR, USA) used for all pelvic binders in this study.	<u>30-d mortality, n/N (%)</u><br>IG: 0/20 (0) vs. CG: 1/23 (2.3), p=0.99<br><u>Blood product transfusion, n/N (%)</u><br>IG: 1/20 (5) vs. CG: 5/23 (21.7), p=0.19<br><u>Surgical control of pelvic hemorrhage, n/N (%)</u><br>IG: 0/20 (0) vs. CG: 0/23 (0)<br><u>Angioembolization for pelvic hemorrhage, n/N (%)</u><br>IG: 2/20 (10) vs. CG: 3/23 (13.0), p=0.99<br><u>30-d readmission, n/N (%)</u><br>IG: 1/20 (5.0) vs. CG: 2/23 (8.7), p=0.99	**Level of evidence**<br>2b ↓<br><br>**Risk of bias**<br>Selection bias: +<br>Performance bias: –<br>Attrition bias: ?<br>Detection bias: +<br><br>**Authors' conclusion**<br>"80% of binders were placed appropriately without secondary injury to any patient. Further, the model for conducting a prospective, randomized efficacy trial in a prehospital setting was successfully piloted. However, identifying the impact of prehospital pelvic compression device placement on morbidity and mortality would

↓ indicates that the level of evidence was lowered due to concerns related to the study design, as detailed in the Reviewers'conclusion

Study: Reference, aim, design, setting	Participants: selection criteria, characteristics	N Participants; Intervention (IG) vs. Control group (CG)	Main outcomes	Assessment: LoE, risk of bias; Conclusions
placement in the prehospital setting."<br>**Setting**<br>USA, two-year period, years n.r.	Male, n/N (%)<br>IG: 15/20 (75.0) vs. CG: 15/23 (65.2), p=0.53<br>BMI, median<br>IG: 26.9 vs. CG: 23.8, p=0.02<br>ISS ≥16, n/N (%)<br>IG: 6 (30.0) vs. CG: 12 (52.2), p=0.22<br>Pelvic injury, n/N (%)<br>IG: 2 (10.0) vs. CG: 7 (30.4), p=0.14<br>Met inclusion criteria, n/N (%)<br>IG: 8 (36.4) vs. CG: 17 (60.7), p=0.09			require a much larger patient cohort."<br>**Reviewers' conclusion**<br>The study was a small pilot RCT, and seriously underpowered to draw any meaningful efficacy or safety conclusions. Only 20% of patients had a pelvic injury.
**Pizanis (2013)**<br>"Emergency stabilization of the pelvic ring: Clinical comparison between three different techniques". *Injury, Int. J. Care Injured* 2013; 44: 1760-1764.<br>**Study design**<br>Comparative registry study<br>(German Pelvic Trauma Registry)<br>**Aim of the study**<br>"The aim of the present study was to compare (i) demography, (ii) pattern and severity of injuries, (iii) time between admission and procedure,	**Inclusion criteria**<br>• patients with fractures or disruptions of the pelvic ring<br>• treated by circumferential sheets, binders, or c-clamps<br>**Exclusion criteria**<br>• patients who received a combination of different emergency stabilization measures<br>**Characteristics**<br>Age [y], median<br>sheet: 47<br>binder: 26<br>c-clamp: 42, p=0.01<br>Female, n (%)<br>sheet: 5 (16)<br>binder: 10 (36)<br>c-clamp: 46 (35), p=0.12	**Participants**<br>N=192 patients<br>**Study groups**<br>sheet: circumferential sheets; time of application unclear (N=31)<br>binder: circumferential binders; time of application unclear (N=28)<br>c-clamp: c-clamps; time of application unclear (N=133)<br>Fifteen patients who received a combination of different emergency stabilization measures were excluded from the analysis.<br>**Adjusting variables in multivariate logistic regression**<br>• age (by year)<br>• additional packing (yes vs. no)<br>• ISS (by ISS point)	**Adjusted outcomes**<br>Mortality, OR (95% CI)<br>sheet: 3.26 (1.15-9.26), p=0.03<br>c-clamp: reference<br>**Unadjusted outcomes**<br>Mortality, %$<br>sheet: 40<br>binder: 22<br>c-clamp: 21, p=0.08<br>Lethal bleeding from the pelvic region, %$<br>sheet: 23<br>binder: 4<br>c-clamp: 8, p=0.02<br>$ extracted graphically	**Level of evidence**<br>2b<br>**Risk of bias**<br>Selection bias: +<br>Performance bias: ?<br>Attrition bias: +<br>Detection bias: +<br>**Authors' conclusion**<br>"Our data suggest that emergency stabilization of the pelvic ring by c-clamps in younger patients with lower ISS is associated with less mortality. Unadjusted analyses showed a lower rate of lethal pelvic bleeding for binders and c-clamps in comparison with

Study: Reference, aim, design, setting	Participants: selection criteria, characteristics	N Participants; Intervention (IG) vs. Control group (CG)	Main outcomes	Assessment: LoE, risk of bias; Conclusions
(iv) additional emergency measures, (v) transfusion requirement of packed red blood cells, (vi) length of hospital stay, (vii) mortality, and (viii) incidence of lethal pelvic bleeding between patients, which were treated by circumferential sheets, binders, and c-clamps for emergency stabilization of the pelvic ring.“<br>**Setting**<br>Germany, 2004-2012	ISS, median (IQR)<br>sheet: 34 (29-50)<br>binder: 34 (22-41)<br>c-clamp: 36 (29-48), p=0.30<br>SBP [mmHg], median (IQR)<br>sheet: 80 (60-110)<br>binder: 90 (80-120)<br>c-clamp: 90 (75-100), p=0.43			sheet wrapping. Circumferential sheets and binders seem to be, however, faster applicable than the c-clamp.“<br>**Reviewers’ conclusion**<br>The study results need to be interpreted with caution due to the retrospective study design. The groups were not balanced at baseline, but the analysis was adjusted for important confounders. An adjusted analysis for mortality is not reported for circumferential binders.
**Schweigkofler (2021)**<br>"Is there any benefit in the pre-hospital application of pelvic binders in patients with suspected pelvic injuries?" *European Journal of Trauma and Emergency Surgery* 2021; 47: 493-498.<br>**Study design**<br>Prospective observational multi-center study (subgroup analysis)<br>**Aim of the study**<br>“The aim of this study was to evaluate the clinical effect of an early pelvic	**Inclusion criteria**<br>• all patients admitted to the emergency room of the BG Unfallklinik Frankfurt am Main<br>• radiologically confirmed type B or C (according to Tile) pelvic ring fracture<br>• blood requirement in the first 72 h after admission<br>**Exclusion criteria**<br>• patients transferred from another hospital<br>**Characteristics of patients with pRBC req.**<br>Age [y], mean ± SD (range)<br>IG: 51 ± 19.6 (16–88)<br>CG: 48 ± 21.0 (17–78), p=0.402	**Participants**<br>N=64 patients with B/C pelvic ring fracture, 35 patients with pRBC requirement<br>**Study groups**<br>IG: pelvic binder applied during prehospital treatment (N=37 overall, N=20 with pRBC requirement)<br>CG: no pelvic binder (N=27 overall, N=15 with pRBC requirement)	**Adjusted outcomes**<br>Mortality, SMR§<br>IG: 1.06 vs. CG: 1.35, p=0.500<br>**Unadjusted outcomes**<br>Mortality, n/N (%)<br>IG: 4/20 (20)<br>CG: 2/15 (13.33), p=0.452<br>pRBC in 72h [units], mean ± SD (range)<br>IG: 10.5 ± 7.8 (1–30)<br>CG: 7.5 ± 8.4 (1–35), p=0.457<br>Mass transfusion (≥10 pRBC/24 h), n/N (%)<br>IG: 7/20 (35)<br>CG: 3/15 (20), p=0.247	**Level of evidence**<br>3b↓<br>**Risk of bias**<br>Selection bias: –<br>Performance bias: ?<br>Attrition bias: +<br>Detection bias: +<br>**Authors’ conclusion**<br>“We were unable to identify blood-saving effects with application of a pelvic binder to patients with instable pelvic ring fractures in terms of RPBC requirements.

Study: Reference, aim, design, setting	Participants: selection criteria, characteristics	N Participants; Intervention (IG) vs. Control group (CG)	Main outcomes	Assessment: LoE, risk of bias; Conclusions
binder application in multiple trauma patients with suspected pelvic fracture (as a potential bleeding source) on patients outcome and transfusion requirements.“<br><br>**Setting**<br><br>Germany, 2013-2014	<u>Sex, male, n (%)</u><br>IG: 12 (60)<br>CG: 9 (60), p=0.637<br><br><u>ISS, mean ± SD</u><br>IG: 29.7 ± 12.3<br>CG: 24.4 ± 9.0, p=0.082<br><br><u>NISS, mean ± SD</u><br>IG: 35.2 ± 14.1<br>CG: 31.3 ± 10.2, p=0.323<br><br><u>TASH on admission, mean ± SD</u><br>IG: 10.1 ± 5.7<br>CG: 6.2 ± 3.9, p=0.690<br><br><u>RISC II survival probability [%], mean ± SD</u><br>IG: 81.2 ± 22.9<br>CG: 89.2 ± 15.2, p=0.525<br><br><u>Rate of abdominal injury, AIS>3, n (%)</u><br>IG: 6 (16.2)<br>CG: 2 (7.4)<br><br><u>Severe TBI, AIS>3, n (%)</u><br>IG: 5 (13.5)<br>CG: 5 (18.5), p=0.589<br><br><u>Isolated pelvic injury, n (%)</u><br>IG: 2 (5.4)<br>CG: 2 (7.4), p=0.759		[§] standardized mortality rate (SMR): observed divided by expected mortality, using the Revised Injury Severity Classification Score II (RISC-II) to estimate probability of survival	Nevertheless, some salutary effect of prehospital pelvic binder application may be assumed.“<br><br>**Reviewers' conclusion**<br><br>This was a post-hoc subgroup analysis with very small sample size, and probably underpowered to detect differences in either baseline characteristics or outcomes.

+: low risk; –: high risk; ?: unclear risk; AIS: Abbreviated Injury Scale; BMI: body mass index; CI: Confidence Interval; GCS: Glasgow Coma Scale; ED: emergency department; HR: Hazard Ratio; ICU: intensive care unit; IQR: interquartile range; ISS: injury severity score; ITT: Intention to Treat; NISS: new injury severity score; OR: Odds Ratio; PCCD: pelvic circumferential compression device; PH: prehospital; pRBC: packed red blood cells; req.: requirement; RISC-II: Revised Injury Severity Classification Score II; RR: Relative Risk; SBP: systolic blood pressure; SD: Standard Deviation; SEM: Standard Error of Mean; SMR: standardized mortality rate; TASH: Trauma Associated Severe Hemorrhage Score; TBI: traumatic brain injury. adj.: adjusted; d: days; m: months; y: years; n.r.: not reported

Compression dressings

Study: Reference, aim, design, setting	Participants: selection criteria, characteristics	N Participants; Intervention (IG) vs. Control group (CG)	Main outcomes	Assessment: LoE, risk of bias; Conclusions
**Taghavi (2021)**<br>"An Eastern Association for the Surgery of Trauma multicenter trial examining prehospital procedures in penetrating trauma patients" *J Trauma Acute Care Surg* 2021; 91(1): 130-140.<br>**Study design**<br>Prospective cohort study<br>(Eastern Association for the Surgery of Trauma)<br>**Aim of the study**<br>"The goal of this study was to evaluate the influence of PHPs on outcomes in penetrating trauma patients in urban locations."<br>**Setting**<br>USA (urban), 2019-2020	**Inclusion criteria**<br>• adults (≥18 y) with penetrating trauma<br>• gunshot or stab wound to the torso and/or proximal extremity<br>• torso and/or proximal extremity penetrating injury combined with distal extremity penetrating injury<br>• penetrating torso and/or proximal extremity injury combined with a blunt injury<br>**Exclusion criteria**<br>• patients with isolated injury above the clavicle (including head or neck [including TBI]),<br>• distal extremity injury only (distal to elbows or knees),<br>• isolated blunt mechanism of injury<br>• patients transferred from outside institutions<br>• known age ≤17 y<br>**Characteristics**[§]<br><u>Age [y], mean ± SD</u><br>PHP: 33.3 ± 12.9<br>CG: 31.2 ± 11.6, p<0.001<br><u>Male, n (%)</u><br>PHP: 1,183 (86.1)<br>CG: 803 (89.5), p=0.02<br><u>NISS, mean ± SD</u><br>PHP: 16 ± 18<br>CG: 12 ± 16, p<0.001	**Participants**<br>N=2,284 patients<br>**Study groups**<br>IG: pressure dressing application (N=409) applied on-scene (N=325) and/or during transport (N=161)<br>CG: no prehospital procedures (N=898)<br>PHP: prehospital procedures including IV access, intraosseous access, fluid administration, bladder catheterization, endotracheal intubation, cervical spine immobilization, pleural decompression, tourniquet placement, pressure dressing application, cricothyrotomy, and pelvic stabilization (N=1,386)<br>**Adjusting variables in multivariate logistic regression**<br>• Age<br>• NISS<br>• gunshot wound<br>• chest injury<br>• higher PH SBP<br>• PH intubation<br>• PH IO access<br>• PH IV placement<br>• PH fluids<br>• PH C-spine immobilization<br>• PH tourniquet<br>• PH pleural decompression	**Adjusted outcomes**<br><u>In-hospital mortality, adj. OR (95% CI)</u><br>IG: 0.80 (0.34–1.87), p=0.60<br>CG: reference<br>**Unadjusted outcomes**<br><u>In-hospital mortality, OR (95% CI)</u><br>IG: 0.58 (0.38–0.90), p=0.01<br>CG: reference	**Level of evidence**<br>2b<br>**Risk of bias**<br>Selection bias: –<br>Performance bias: ?<br>Attrition bias: +<br>Detection bias: +<br>**Authors' conclusion**<br>"Prehospital use of tourniquets and pressure dressings were not associated with benefit on adjusted analysis."<br>**Reviewers' conclusion**<br>The study results need to be interpreted with caution due to the retrospective study design and risk of selection bias. No information on baseline characteristics is provided in the pressure dressing group. The analysis was adjusted for important confounders.

Study: Reference, aim, design, setting	Participants: selection criteria, characteristics	N Participants; Intervention (IG) vs. Control group (CG)	Main outcomes	Assessment: LoE, risk of bias; Conclusions
	<u>GCS, mean ± SD</u><br>PHP: 13.7 ± 3.5<br>CG: 14.3 ± 2.6, p=0.02<br><u>Shock index (HR/SBP)</u><br>PHP: 0.8 ± 0.4<br>CG: 0.7 ± 0.2, p=0.02<br>§ characteristics not reported for IG (pressure dressings) separately from other PH procedures			

+: low risk; –: high risk; ?: unclear risk; CI: Confidence Interval; HR: Hazard Ratio; IO: intraosseous; IQR: Interquartile Range; ISS: injury severity score; ITT: Intention to Treat; NISS: new injury severity score; OR: Odds Ratio; PH: prehospital; pRBC: packed red blood cells; RISC-II: Revised Injury Severity Classification Score II; RR: Relative Risk; SBP: systolic blood pressure; SD: Standard Deviation; SEM: Standard Error of Mean; SMR: standardized mortality rate; TASH: Trauma Associated Severe Hemorrhage Score; TBI: traumatic brain injury. adj.: adjusted; d: days; m: months; y: years

Tourniquets

Study: Reference, aim, design, setting	Participants: selection criteria, characteristics	N Participants; Intervention (IG) vs. Control group (CG)	Main outcomes	Assessment: LoE, risk of bias; Conclusions
**Clasper (2009)**<br>"Limb complications following pre-hospital tourniquet use". *J R Army Med Corps*. 2009; 155(3): 200-202.<br>**Study design**<br>Comparative registry study<br>(Joint Theatre Trauma Register)<br>**Aim of the study**<br>"The aim of this study was to investigate if the pre-hospital application of a	**Inclusion criteria**<br>• AIS >1 in the lower limbs<br>• lower limb injury with fracture<br>**Exclusion criteria**<br>n.r.<br>**Characteristics**<br><u>Age [y], mean (range)</u><br>IG: 26.6 (19-37) vs. CG: 25.7 (19-37)<br><u>ISS, median</u><br>IG: 10 vs. CG: 10<br><u>MESS, median</u><br>IG: 5 vs. CG: 5	**Participants**<br>N=58 limbs<br>**Study groups**<br>IG: tourniquet used (N=23 limbs, N=22 after matching)<br>19 tourniquets were applied for a median of 60 min [range 19-150 min], for 3 it was impossible to determine accurately the applied tourniquet time. 3 were applied for ≥120 min (currently recommended maximum time).<br>CG: no tourniquet used (N=35 limbs, N=22 after matching)<br>**Matching**	<u>Total number of limbs with any complication, n/N</u><br>IG: 19/22 vs. CG: 15/22, p=0.13<br><u>Superficial wound infection, n</u><br>IG: 11/22 vs. CG: 11/22, NS<br><u>Major complications, n</u><br>IG: 10/22 vs. CG: 4/22, p=0.045<br><u>Failed salvage [amputation required], n</u><br>IG: 3/22 vs. CG: 3/22, NS<br><u>Deep infection [osteomyelitis], n</u><br>IG: 7/22 [4/22] vs. CG: 1/22 [0/22], p<0.05<br><u>Flap failure, n</u><br>IG: 1/22 vs. CG: 0/22, NS	**Level of evidence**<br>3b⇃<br>**Risk of bias**<br>Selection bias: –<br>Performance bias: –<br>Attrition bias: +<br>Detection bias: +<br>**Authors' conclusion**<br>"Ultimately the use of the tourniquet may have saved lives, and did not increase the amputation rate in this small study, and so despite

Study: Reference, aim, design, setting	Participants: selection criteria, characteristics	N Participants; Intervention (IG) vs. Control group (CG)	Main outcomes	Assessment: LoE, risk of bias; Conclusions
tourniquet resulted in an increase in morbidity following significant ballistic limb injury."<br><br>**Setting**<br><br>UK military: Afghanistan / Iraq, 2003-2008	Time to initial surgery >6 h from injury, n<br><br>IG: 4 vs. CG: 4<br><br>Bone involved, n<br><br>*Femur* IG: 6 vs. CG: 7<br><br>*Patella* IG: 1 vs. CG: 1<br><br>*Tibia* IG: 10 vs. CG: 11<br><br>*Isolated fibula* IG: 1 vs. CG: 1<br><br>*Ankle + Hindfoot* IG: 2 vs. CG: 1<br><br>Hindfoot/Midfoot IG: 2 vs. CG: 1	An experienced military orthopaedic surgeon blinded to the study (PH) matched each casualty from the pre-hospital tourniquet group with a casualty from the prehospital non-tourniquet group; all data was anonymised and identifiable by JTTR number only. Although the exact details of the matching were left to the surgeon, each casualty was only used once and matched for anatomical location, severity of the bony injury, initial surgical management, ISS and MESS as much as possible.		the increased deep infection rate the use of pre-hospital tourniquets cannot be decried as a result of this study."<br><br>**Reviewers' conclusion**<br><br>The study results need to be interpreted with caution due to risk of selection and performance bias. The study is very small and details of matching were left to the surgeon. Only 43% of patients were severely injured.
**Henry (2021)**<br><br>"Increased Use of Prehospital Tourniquet and Patient Survival: Los Angeles Countywide Study" *J Am Coll Surg*, 2021; 233(2): 233-239.e2.<br><br>**Study design**<br><br>Comparative registry study<br><br>(LA County Department of Health Services EMS provider registry and trauma registry)<br><br>**Aim of the study**<br><br>"to determine whether the use of tourniquets in Los Angeles County (LAC) has been increasing over	**Inclusion criteria**<br><br>• Patients with peripheral arterial injuries<br>• transported by EMS to 1 of 15 Level I or II trauma centers<br><br>**Exclusion criteria**<br><br>NR<br><br>**Characteristics**<br><br>Age [y], mean ± SD<br><br>IG: 34.8 ± 13.3 vs. CG: 36.8 ± 12.4, p=0.201<br><br>Male, n (%)<br><br>IG: 83 (85.6) vs. CG: 712 (84.1), p=0.674<br><br>ISS, mean ± SD<br><br>IG: 13.4 ± 8.1 vs. CG: 13.7 ± 7.3, p=0.104<br><br>GCS, mean ± SD	**Participants**<br><br>N=944 patients<br><br>**Study groups**<br><br>IG: prehospital tourniquet used (N=97)<br><br>CG: no prehospital tourniquet used (N=847)<br><br>The decision to place a tourniquet was made by the prehospital EMS provider and is not standardized across the county.<br><br>**Adjusting variables in multivariable regression analysis**<br><br>• penetrating mechanism<br>• traumatic amputations<br>• ISS<br>• prehospital transport time<br>• prehospital heart rate >100 beats/min<br>• prehospital SBP <90 mmHg<br>• prehospital GCS <9	**Adjusted outcomes**<br><br>In-hospital mortality, adj. OR (95% CI)*<br><br>0.32 (0.16 to 0.85), p=0.032<br><br>Delayed amputation, adjusted OR (95% CI)*<br><br>1.07 (0.21 to 10.88), p=0.097<br><br>ICU LOS [d], (95% CI)*<br><br>-0.18 (-1.74 to 0.11), p=0.799<br><br>PRBC transfusion 4-h volume (mL], (95% CI)*<br><br>-547.76 (-762.73 to -283.49), p<0.001<br><br>PRBC transfusion 24-h volume [mL] (95% CI)*<br><br>-1,389.82 (-1,824.88 to -920.97), p<0.001<br><br>* prehospital tourniquet vs no prehospital tourniquet<br><br>**Unadjusted outcomes**<br><br>In-hospital mortality, n (%)	**Level of evidence**<br><br>2b<br><br>**Risk of bias**<br><br>Selection bias: –<br><br>Performance bias: –<br><br>Attrition bias: +<br><br>Detection bias: +<br><br>**Authors' conclusion**<br><br>"The use of prehospital tourniquets for patients with extremity vascular injuries is significantly associated with improved survival and decreased blood transfusion requirement, without an

Study: Reference, aim, design, setting	Participants: selection criteria, characteristics	N Participants; Intervention (IG) vs. Control group (CG)	Main outcomes	Assessment: LoE, risk of bias; Conclusions
time and whether prehospital tourniquet use is associated with improved patient outcomes without complications."<br>**Setting**<br>USA, 2015-2019	IG: 13 ± 1.9 vs. CG: 13 ± 2.3, p=0.753<br>SBP [mmHg], mean ± SD<br>IG: 113 ± 45.4 vs. CG: 119 ± 33.2, p=0.055<br>Extremity AIS ≥4, n (%)<br>IG: 24 (24.7) vs. CG: 86 (10.2), p=0.004<br>Traumatic amputation, n (%)<br>IG: 1 (1.0) vs. CG: 27 (3.2), p=0.087		IG: 1 (1.0) vs. CG: 75 (8.9), p=0.027<br>Delayed amputation, n (%)<br>IG: 6 (0.7) vs. CG: 1 (1.0), p=0.727<br>ICU LOS [d], median (IQR)<br>IG: 4.2 (1.9-6.2) vs. CG: 3.9 (2.2-7.1), p=0.868<br>Transfusion 4-h PRBC volume [mL], median (IQR)<br>IG: 462.8 (107.3-749.7) vs. CG: 1041.3 (682.2-2674.9), p<0.001<br>Transfusion 24-h PRBC volume [mL], median (IQR)<br>IG: 994.6 (559.4-1304.1) vs. CG: 2469.1 (981.2-5117.5), p<0.001	increased risk of delayed amputation."<br>**Reviewers' conclusion**<br>The study results need to be interpreted with caution due to risk of selection and performance bias. Patients in the tourniquet group had significantly higher extremity AIS. Patients did not fulfil the criteria of polytrauma (ISS≥15, multiple injuries).
**Kauvar (2018)**<br>"Tourniquet use is not associated with limb loss following military lower extremity arterial trauma". *J Trauma Acute Care Surg.* 2018; 85(3): 495-499.<br>**Study design**<br>Comparative registry study<br>(Department of Defense Trauma Registry)<br>**Aim of the study**<br>"The purpose of this study was to use a military lower extremity vascular injury database with long-term follow-up to study the influence of	**Inclusion criteria**<br>• limbs sustaining at least one arterial injury to the common, superficial, or deep femoral, popliteal, or tibial arteries<br>• undergoing at least one limb salvage procedure in the OIF or OEF theaters of operations<br>**Exclusion criteria**<br>• Casualties sustaining traumatic amputations and those with vascular injuries managed with amputation at the index operation<br>**Characteristics**<br>Age [y], mean ± SD<br>IG: 27 ± 7 vs. CG: 26 ± 6, NS<br>ISS, mean ± SD<br>IG: 17 ± 9 vs. CG 16 ± 10, NS	**Participants**<br>N=455 limbs<br>**Study groups**<br>IG: extremity tourniquet use (N=254 limbs)<br>CG: no extremity tourniquet use (N=201 limbs)<br>**Co-interventions**<br>Recombinant factor VIIa and/or TXA, n (%)<br>IG:48 (19) vs. CG: 22 (11), p=0.02	**Systemic outcomes, unadjusted**<br>Mortality, n (%)<br>IG: 8 (3.2) vs. CG: 8 (4.0), NS<br>Rhabdomyolysis, n (%)<br>IG: 26 (10) vs. CG: 18 (9.0), NS<br>Pulmonary embolism, n (%)<br>IG: 18 (7.1) vs. CG: 5 (2.5), p=0.026<br>Whole blood + PRBC, mean ± SD<br>IG: 31 ± 26 vs. CG: 25 ± 19, NS<br>Arterial shunt, n (%)<br>IG: 54 (21) vs. CG: 30 (15), p=0.08<br>Arterial bypass, n (%)<br>IG: 122 (48) vs. CG: 92 (46), p=NS<br>Fasciotomy, n (%)<br>IG: 195 (77) vs. CG: 134 (67), p=0.02	**Level of evidence**<br>2b<br>**Risk of bias**<br>Selection bias: –<br>Performance bias: –<br>Attrition bias: +<br>Detection bias: +<br>**Authors' conclusion**<br>"In combat-related lower extremity trauma with arterial injury, tourniquet use before initial surgical care was not associated with early or eventual limb loss despite increased limb injury severity. Tourniquet use was associated with some eventual adverse

Study: Reference, aim, design, setting	Participants: selection criteria, characteristics	N Participants; Intervention (IG) vs. Control group (CG)	Main outcomes	Assessment: LoE, risk of bias; Conclusions
tourniquet use on long-term limb outcomes following arterial injury."<br>**Setting**<br>US military/Iraq, 2004-2012	AIS extremity, median (range)<br>IG: 3 (3–4) vs. CG: 3 (3–3.5), p=0.02<br>MESS, median (range)<br>IG: 6 (5–7) vs. CG: 6 (5–7), p=0.006<br>Fracture, n (%)<br>IG: 153 (60) vs. CG: 112 (56), NS<br>Nerve injury, n (%)<br>IG: 144 (57) vs. CG: 91 (45), p=0.015<br>Vascular injury above the knee, n (%)<br>IG: 145 (57) vs. CG: 109 (54), NS<br>Venous injury, n (%)<br>IG: 50 (20) vs. CG: 43 (21), NS		**Limb complications, unadjusted**<br>Amputation, n (%)<br>IG: 63 (25) vs. CG: 40 (19), NS<br>Amputation above the knee, n (%)<br>IG: 28 (11), vs. CG: 11 (5.4), p=0.11<br>Vascular repair, n (%)<br>IG: 51 (20) vs. CG: 27 (13), p=0.06<br>Wound infection, n (%)<br>IG: 79 (31) vs. CG: 41 (20), p=0.01<br>Contracture, n (%)<br>IG: 21 (8.3) vs. CG: 11 (5.5), NS<br>Foot drop, n (%)<br>IG: 70 (28) vs. CG: 35 (17), p=0.011<br>Sensory deficit, n (%)<br>IG: 67 (26) vs. CG: 63 (31), NS<br>Severe edema, n (%)<br>IG: 108 (43) vs. CG: 70 (35), NS<br>Deep venous thrombosis, n (%)<br>IG: 21 (8.3) vs. CG: 12 (6.0), NS	limb outcomes, however, indicating that tourniquets should continue to be used for well-defined indications and rapid surgical control of limb hemorrhage should remain a priority during modern military operations."<br>**Reviewers' conclusion**<br>There is a risk of performance bias as less patients in the intervention group were treated in a role 2 (level of surgical care) institution. Moreover, the authors state that they are unaware of the initial treatment except for the tourniquet. The groups differ with respect to important risk factors, and the results are unadjusted.
**Kragh (2015)**<br>"U.S. Military use of tourniquets from 2001 to 2010". *Prehospital Emergency Care* 2015; 19(2): 184–190.<br>**Study design**	**Inclusion criteria**<br>• active-duty casualties at any U.S. military hospital in either Afghanistan or Iraq<br>• major limb trauma, extremity AIS ≥3<br>• extremity AIS 1 to 5 if paired with an associated external AIS ≥3<br>**Exclusion criteria**	**Participants**<br>N=4,297 patients<br>**Study groups**<br>IG: extremity tourniquet used (N=1,272)<br>CG: no extremity tourniquet used (N=3,025)	Mortality, n/N (%)<br>IG: 102/1272 (8.0)<br>CG: 112/3025 (3.7), p<0.0001	**Level of evidence**<br>3b↓<br>**Risk of bias**<br>Selection bias: –<br>Performance bias: ?<br>Attrition bias: +

Study: Reference, aim, design, setting	Participants: selection criteria, characteristics	N Participants; Intervention (IG) vs. Control group (CG)	Main outcomes	Assessment: LoE, risk of bias; Conclusions
Comparative registry study<br><br>(Department of Defense Trauma Registry)<br><br>**Aim of the study**<br><br>"The purpose of the present study is to associate tourniquet use and survival in casualty care over a decade in order to provide evidence to emergency medical personnel for the implementation and efficacy of tourniquet use in a large trauma system."<br><br>**Setting**<br><br>US military: Afghanistan / Iraq, 2001-2010	• casualties killed prior to reaching hospital, or who arrive without vital signs (and are not regained)<br><br>**Characteristics**<br><br><u>Age, median (range)</u><br><br>24 (18-60)<br><br><u>Male, n (%)</u><br><br>IG: 1,250 (98.2) vs. CG: 2,948 (97.5)			Detection bias: +<br><br>**Authors' conclusion**<br><br>"In summary, the present survey of war casualties with extremity injury shows that survival rates are increased in those casualties with injuries amenable to tourniquet use, despite an increased injury severity. The findings of the present study are 1) tourniquet use rates have risen in recent years; 2), survival rates of those casualties with injuries amenable to tourniquet use rose concurrently; 3) those with injuries that were not amenable to tourniquet use decreased; and 4) tourniquet requirement rates are opportunities for improvement."<br><br>**Reviewers' conclusion**<br><br>There is a high risk of selection bias due to the exclusion of casualties who arrived dead at the hospital and unclear timing of tourniquet placement. No information is provided on injury severity by group. The results are unadjusted and groups may differ

Study: Reference, aim, design, setting	Participants: selection criteria, characteristics	N Participants; Intervention (IG) vs. Control group (CG)	Main outcomes	Assessment: LoE, risk of bias; Conclusions
				regarding important confounders.<br>The population may partially overlap with Kauvar (2018).
**Kragh (2015)**<br>"Transfusion for Shock in US Military War Casualties With and Without Tourniquet Use". *Ann Emerg Med.* 2015; 65(3): 290-296.<br>**Study design**<br>Comparative registry study<br>(Department of Defense Trauma Registry)<br>**Aim of the study**<br>"The purpose of the present study of transfused war casualties admitted to US military hospitals is to determine whether there were any associations among severities of injury, tourniquet use, and survival to better understand the effect of tourniquet use on outcomes in hemorrhagic shock."<br>**Setting**	**Inclusion criteria**<br>• active-duty casualties who arrived alive at any US military hospital<br>• major limb trauma AIS (upper/lower extremities) >2<br>• transfusion of a blood product<br>• tourniquet use<br>**Exclusion criteria**<br>• detainees, prisoners<br>• died on arrival or before arrival to the first military hospital<br>**Characteristics**<br>Age [y], mean ± SD<br>IG: 26 ± 6.1 vs. CG: 26 ± 6.1<br>ISS, mean ± SD; MD (95% CI)<br>IG: 19 ± 10.6 vs. CG 21 ± 11.8<br>MD 2 (0.65-2.99)<br>SBP [mmHg], mean ± SD<br>IG: 111 ± 37 vs. CG: 112 ± 33.7<br>AIS extremity, n (%)<br>Serious: IG: 465 (64.6) vs. CG: 555 (80.1)<br>Severe: IG: 248 (34.4) vs. CG: 111 (16)<br>Critical: IG: 7 (1) vs. CG: 27 (3.9)<br>GCS, mean ± SD	**Participants**<br>N=1,413 patients, 502 after propensity matching<br>**Study groups**<br>IG: extremity tourniquet used (N=720 total, N=251 after matching)<br>CG: no extremity tourniquet used (N=693 total, N=251 after matching)<br>**Matching criteria**<br>• admission hemoglobin, pulse rate, adjusted ISS, extremity AIS score<br>• casualties who had only minor or no injuries of the head, face, chest, and abdomen; this second propensity matching was intended to remove casualties with major injuries that would not benefit from tourniquet use.	**Outcomes after propensity matching**<br>Mortality, %, MD; OR (95% CI)<br>IG: 6.8 vs. CG: 8.8, MD 2, p=0.40<br>OR 0.916 (0.450-1.865)<br>Casualties who had only minor or no injuries of the head, face, chest, and abdomen:<br>Mortality, %, MD<br>IG: 3.4 (N=207)<br>CG: 5.5 (N=207), MD 2.1, p=0.40<br>**Outcomes, unadjusted**<br>Survival, n/N (%)<br>IG: 632/720 (88) vs. CG: 614/693 (89), p=0.62<br>Red blood cells [units], mean ± SD, MD (95% CI)<br>IG: 12 ± 11.4 vs. CG: 9 ± 8.8, MD -3 (-4.97 to -2.85)<br>Platelets [units], mean ± SD, MD (95% CI)<br>IG: 1 ± 2.0 vs. CG: 0.5 ± 1.9, MD -0.5 (-0.71 to -0.31)<br>Cryoprecipitate [units], mean ± SD, MD (95% CI)<br>IG: 0.4 ± 1.07 vs. CG: 0.3 ± 0.9, MD -0.1 (-0.21 to 0)<br>Whole blood [units], mean ± SD, MD (95% CI)<br>IG: 2 ± 5.3 vs. CG: 1 ± 3.9, MD -1 (-0.86 to 0.11)<br>Plasma [units], mean ± SD, MD (95% CI)	**Level of evidence**<br>2b<br>**Risk of bias**<br>Selection bias: –<br>Performance bias: +<br>Attrition bias: +<br>Detection bias: +<br>**Authors' conclusion**<br>"Given the data available to us today, we see no design option that alters the likelihood of detecting a survival benefit with tourniquet use in a registry that excludes out-of-hospital deaths. Although tourniquets may appear to be a proven solution for hemorrhage control, the science is limited and research needs to be conducted."<br>**Reviewers' conclusion**<br>The authors' conclusions account for the high risk of selection bias due to the exclusion of casualties who

Study: Reference, aim, design, setting	Participants: selection criteria, characteristics	N Participants; Intervention (IG) vs. Control group (CG)	Main outcomes	Assessment: LoE, risk of bias; Conclusions
US military, Afghanistan / Iraq, 2001-2008	IG: 13 ± 4.3 vs. CG: 13 ± 4.3		IG: 7 ± 9.2 vs. CG: 5 ± 7.2, MD -2 (-3.40 to -1.68)<br><u>Sum of blood products, mean ± SD, MD (95% CI)</u><br>IG: 14 ± 14.4 vs. CG: 10 ± 10.8, MD -4 (-5.60 to -2.96)<br><u>Total ICU length of stay [d], mean ± SD, MD (95% CI)</u><br>IG: 7 ± 13.8 vs. CG 8 ± 13.7, MD 1 (-0.45 to 2.42)<br><u>Total length of stay [d], mean ± SD, MD (95% CI)</u><br>G: 34 ± 31.4 vs. CG 33 ± 35.6, MD 1 (-4.15 to 2.87)IG: 6.8 vs.CG: 8.8, MD 2, p=0.	arrived dead at the hospital. The mortality outcome, though adjusted for measured confounders, may be strongly biased favouring the control group without tourniquet placement.<br>The population likely overlaps with Kragh (2015) and Kauvar (2018).
**Taghavi (2021)**<br>"An Eastern Association for the Surgery of Trauma multicenter trial examining prehospital procedures in penetrating trauma patients" *J Trauma Acute Care Surg* 2021; 91(1): 130-140.<br>**Study design**<br>Prospective cohort study<br>(Eastern Association for the Surgery of Trauma)<br>**Aim of the study**<br>"The goal of this study was to evaluate the influence of PHPs on outcomes in penetrating trauma patients in urban locations."<br>**Setting**	**Inclusion criteria**<br>• adults (≥18 y) with penetrating trauma<br>• gunshot or stab wound to the torso and/or proximal extremity<br>• torso and/or proximal extremity penetrating injury combined with distal extremity penetrating injury<br>• penetrating torso and/or proximal extremity injury combined with a blunt injury<br>**Exclusion criteria**<br>• patients with isolated injury above the clavicle (including head or neck [including TBI]),<br>• distal extremity injury only (distal to elbows or knees),<br>• isolated blunt mechanism of injury<br>• patients transferred from outside institutions<br>• known age ≤17 y<br>**Characteristics**[§]<br><u>Age [y], mean ± SD</u>	**Participants**<br>N=2,284 patients<br>**Study groups**<br>IG: tourniquet placement (N=108) applied on-scene (N=86) and/or during transport (N=29)<br>CG: no prehospital procedures (N=898)<br>PHP: prehospital procedures including IV access, intraosseous access, fluid administration, bladder catheterization, endotracheal intubation, cervical spine immobilization, pleural decompression, tourniquet placement, pressure dressing application, cricothyrotomy, and pelvic stabilization (N=1,386)<br>**Adjusting variables in multivariate logistic regression**<br>• Age<br>• NISS<br>• gunshot wound<br>• chest injury	**Adjusted outcomes**<br><u>In-hospital mortality, adj. OR (95% CI)</u><br>IG: 0.70 (0.14–3.93), p=0.65<br>CG: reference<br>**Unadjusted outcomes**<br><u>In-hospital mortality, OR (95% CI)</u><br>IG: 0.66 (0.30–1.44), p=0.30<br>CG: reference	**Level of evidence**<br>2b<br>**Risk of bias**<br>Selection bias: –<br>Performance bias: ?<br>Attrition bias: +<br>Detection bias: +<br>**Authors' conclusion**<br>"Prehospital use of tourniquets and pressure dressings were not associated with benefit on adjusted analysis."<br>**Reviewers' conclusion**<br>The study results need to be interpreted with caution due to the retrospective study design and risk of selection bias. No

Study: Reference, aim, design, setting	Participants: selection criteria, characteristics	N Participants; Intervention (IG) vs. Control group (CG)	Main outcomes	Assessment: LoE, risk of bias; Conclusions
USA (urban), 2019-2020	PHP: 33.3 ± 12.9<br>CG: 31.2 ± 11.6, p<0.001<br><br>Male, n (%)<br>PHP: 1,183 (86.1)<br>CG: 803 (89.5), p=0.02<br><br>NISS, mean ± SD<br>PHP: 16 ± 18<br>CG: 12 ± 16, p<0.001<br><br>GCS, mean ± SD<br>PHP: 13.7 ± 3.5<br>CG: 14.3 ± 2.6, p=0.02<br><br>Shock index (HR/SBP)<br>PHP: 0.8 ± 0.4<br>CG: 0.7 ± 0.2, p=0.02<br><br>§ characteristics not reported for IG (tourniquets) separately from other PH procedures	• higher PH SBP<br>• PH intubation<br>• PH IO access<br>• PH IV placement<br>• PH fluids<br>• PH C-spine immobilization<br>• PH pressure dressing<br>• PH pleural decompression		information on baseline characteristics is provided in the tourniquet group. The analysis was adjusted for important confounders.
+: low risk; –: high risk; ?: unclear risk; AIS: Abbreviated Injury Scale; CI: Confidence Interval; GCS: Glasgow Coma Scale; ED: emergency department; HR: Hazard Ratio; ICU: intensive care unit; IQR: Interquartile Range; ISS: Injury Severity Score; ITT: Intention to Treat; IV: Intravenous; LOS: length of stay; MD: Mean Difference; MESS: Mangled Extremity Severity Score; NISS: new injury severity score; NS: not significant; OR: Odds Ratio; PH: prehospital; PRBC: packed red blood cells; RR: Relative Risk; SBP: systolic blood pressure; SD: Standard Deviation; SEM: Standard Error of Mean; TBI: traumatic brain injury; TXA: tranexamic acid; adj.: adjusted; d: days; m: months; y: years; n.r.: not reported.				

Traction splints

Study: Reference, aim, design, setting	Participants: selection criteria, characteristics	N Participants; Intervention (IG) vs. Control group (CG)	Main outcomes	Assessment: LoE, risk of bias; Conclusions
**Irajpour (2012)**<br>"A comparison between the effects of simple and traction splints on pain intensity in patients with femur fractures". *Iranian J*	**Inclusion criteria**<br>• diagnosed with a closed femoral shaft fracture<br>• aged 15-65 years<br>• not addicted to any drugs	**Participants**<br>N=64 patients<br><br>**Study groups**<br>IG: traction splint (N=32)	**Unadjusted outcomes**<br><br>Pain intensity immediately after splinting [VAS], mean ± SD<br>IG: 6.4 ± 1.2 vs. CG: 6.7 ± 1.4, p=0.441<br><br>Pain intensity 1h after splinting [VAS], mean ± SD	**Level of evidence**<br>2b↓<br><br>**Risk of bias**<br>Selection bias: –

Study: Reference, aim, design, setting	Participants: selection criteria, characteristics	N Participants; Intervention (IG) vs. Control group (CG)	Main outcomes	Assessment: LoE, risk of bias; Conclusions
Nursing Midwifery Res 2012; 17(7): 530-533.<br><br>**Study design**<br><br>Prospective, quasi-experimental study<br><br>**Aim of the study**<br><br>"To determine and compare the impacts of using simple and traction splints on pain intensity of patients with femoral fracture immediately and at the 1st, 6th and 12th h after splinting."<br><br>**Setting**<br><br>Iran, study years n.r.	• had full consciousness when completing the questionnaires<br>• maintaining the splint on the injured organ for ≥12 h after splinting<br>• using morphine sulfate as a painkiller<br><br>**Exclusion criteria**<br><br>• unwilling to continue participation<br>• developed any problems incompatible with the inclusion criteria<br>• did not have the splint on the injured organ for 12 h<br><br>**Characteristics**<br><br><u>Age [y], mean ± SD</u><br><br>IG: 31 ± 14.8 vs. CG: 29 ± 14.1, p=0.547<br><br><u>Male, n (%)</u><br><br>IG: 29 (90.6) vs. CG: 28 (87.5), p=0.698	CG: simple splint (N=32)<br><br>**Co-interventions**<br><br>5 mg of morphine sulfate was used for all patients immediately before splinting.	IG: 4.8 ± 1.0 vs. CG: 6.0 ± 1.3, p=0.0001<br><br><u>Pain intensity 6h after splinting [VAS], mean ± SD</u><br><br>IG: 4.2 ± 1.0 vs. CG: 5.4 ± 0.9, p=0.0001<br><br><u>Pain intensity 12h after splinting [VAS], mean ± SD</u><br><br>IG: 4.0 ± 1.0 vs. CG: 5.1 ± 1.2, p=0.020	Performance bias: –<br><br>Attrition bias: +<br><br>Detection bias: +<br><br>**Authors' conclusion**<br><br>"The significant difference in pain reduction between the simple and traction splint groups at the 1st, 6th, and 12th hour after splinting emphasizes the superiority of traction splints."<br><br>**Reviewers' conclusion**<br><br>The study results need to be interpreted with care considering the substantial risk of selection and performance bias as well as poor reporting. Treatment was in-hospital, and there is no indication of severe injury or polytrauma, so that important considerations in the population of severely injured patients are not accounted for.

+: low risk; –: high risk; ?: unclear risk; n.r.: not reported; SD: Standard Deviation; VAS: visual analogue scale. adj.: adjusted; d: days; m: months; y: years

Hemostatic agents

Study: Reference, aim, design, setting	Participants: selection criteria, characteristics	N Participants; Intervention (IG) vs. Control group (CG)	Main outcomes	Assessment: LoE, risk of bias; Conclusions
**Hatamabadi (2015)**<br>"Celox-Coated Gauze for the Treatment of Civilian Penetrating Trauma: A Randomized Clinical Trial". *Trauma Mon.* 2015; 20(1): e23862<br>**Study design**<br>Randomised controlled trial<br>**Aim of the study**<br>"This trial aimed to evaluate the role of celox in the management of civilian penetrating trauma."<br>**Setting**<br>Iran, 2014	**Inclusion criteria**<br>• age 18-50 years<br>• stab injury to a limb<br>• minimal wound length of 3 cm<br>• bleeding was a concern regardless of the source<br>**Exclusion criteria**<br>• foreign body retained in the wound<br>• history of anticoagulation<br>• required blood products for resuscitation<br>• other hemostatic products used for the control of bleeding in the prehospital setting<br>**Characteristics**<br><u>Age [y], mean ± SD</u><br>IG: 29.99 ± 9.68 vs. CG: 31.01 ± 10.16, p=0.52<br><u>Male, n (%)</u><br>IG: 73 (91.25) vs. CG: 72 (90), p=0.786<br><u>Wound length, n (%)</u><br>*>10 cm* IG: 14 (17.5) vs. CG: 16 (20)<br>*<10 cm* IG: 66 (83.5) vs. CG 64 (80) p=0.685<br><u>Wound depth, n (%)</u><br>*Dermis* IG: 27 (33.75) vs. CG: 33 (41.25)<br>*Facia* IG: 18 (22.5) vs. CG: 25 (31.25)<br>*Muscle* IG: 35 (43.75) vs. CG: 22 (27.5)	**Participants**<br>N=160 patients<br>**Study groups**<br>IG: celox-coated gauze (N=80)<br>CG: regular gauze (N=80)<br>The control group was treated with pressure bandage using a regular 10 × 10 cm gauze, while a celox-coated gauze was used in the intervention group.	<u>Time to control, by strata, n (%)</u><br>*<5 min* IG: 41 (61.19) vs. CG: 26 (38.81)<br>*5 to 10 min* IG: 20 (47.62) vs. CG: 22 (52.38)<br>*≥10 min* IG: 19 (37.25) vs. CG: 32 (62.75)<br>p=0.010<br><u>Number of blood-soaked 10×10 cm gauzes, mean</u><br>IG: 2.63 vs. CG: 3.06, p=0.049<br>**Subgroup analyses**<br>• Stronger association favouring celox-coated gauze in relation to hemostasis among dermal wounds and among wounds with size over 10 cm (p=0.01 and p=0.04, respectively).<br>• no significant association among fascial, muscular, and smaller (<10 cm) wounds<br>• The role of celox in the management of civilian stab wounds in foot seems more efficient (p=0.001)	**Level of evidence**<br>2b↓<br>**Risk of bias**<br>Selection bias: +<br>Performance bias: –<br>Attrition bias: +<br>Detection bias: +<br>**Authors' conclusion**<br>"The results showed that the use of celox-coated gauze reduces the time needed to achieve hemostasis and the amount of blood loss after initiation of the treatment. The challenge is to select patients who gain the most benefit from it."<br>**Reviewers' conclusion**<br>The study results need to be interpreted with caution due to the small number of participants and risk of performance bias. Patients did not fulfil the criteria of polytrauma (ISS≥15, multiple injuries).

Study: Reference, aim, design, setting	Participants: selection criteria, characteristics	N Participants; Intervention (IG) vs. Control group (CG)	Main outcomes	Assessment: LoE, risk of bias; Conclusions
	p=0.095<br><br><u>Prehospital pressure dressing, n (%)</u><br>*By Medics* IG: 28 (35) vs. CG: 34 (42.5)<br>*By Patient* IG: 8 (10) vs. CG: 5 (6.25)<br>*None* IG: 44 (55) vs. CG: 41 (51.25)<br>p=0.28<br><br><u>SBP [cmHg], mean ± SD</u><br>IG: 11.64 ± 1.13 vs. CG: 11.44 ± 1.32<br>p=0.30			
**Kabeer (2019)**<br>"Pre-hospital Hemorrhagic Control Effectiveness of Axiostat® Dressing Versus Conventional Method in Acute Hemorrhage Due to Trauma". *Cureus* 2019; 11(8): e5527<br><br>**Study design**<br>Prospective cohort study<br><br>**Aim of the study**<br>"The objective of this study was to evaluate the safety and efficacy of the chitosan dressing, Axiostat®, in comparison with conventional cotton gauze as a pre-hospital dressing to stop bleeding from scalp wounds."<br><br>**Setting**<br>India, 2012-2013	**Inclusion criteria**<br>• age ≥18 years<br>• bleeding wounds over the scalp<br>• wound size should be covered by a single available size of study device<br><br>**Exclusion criteria**<br>• prior diagnosis of disease or medical condition affecting the ability of blood to clot (e.g., hemophilia)<br>• a non-survivable injury as per the investigator's discretion<br>• patients who, in the opinion of the investigator, may not complete the study for any reason (e.g., patients requiring immediate suturing)<br>• grossly infected wounds which may require multiple debridement procedures prior to clearance of bacteria, and non-viable tissue from the wound<br>• patients currently participating in an investigational drug or device study that had not yet completed its primary	**Participants**<br>N=104<br><br>**Study groups**<br>IG: Axiostat cauted gauze (N=47)<br>Axiostat® is a sterile, single-use, 100% chitosan dressing designed to stop bleeding instantly.<br><br>CG: conventional cotton gauze (N=57)<br><br>The size of the dressing was 8 cm x 5 cm in both groups. If the bleeding was persistent, a second application of the respective dressing was applied.	<u>Time to achieve haemostasis [min], mean ± SD</u><br>IG: 4.68 ± 1.04 vs. CG: 18.56 ± 5.04, p<0.0001<br><br><u>Blood loss [g], mean ± SD</u><br>IG: 5.41 ± 2.53 vs. CG: 11.16 ± 4.96, p<0.0001<br><br><u>Patients with two dressing applications, n/N (%)</u><br>IG: 8/47 (17) vs. CG: 20/57 (35), p=0.039<br><br><u>Number of patients with haemostasis, n/N (%)</u><br>IG: 44/47 (94) vs. CG: 42/57 (74), p=0.007<br><br><u>Side effects (difficulties removing the dressing, tissue loss, rebleeding), n/N</u><br>IG: 0/47 vs. CG: 3/57<br><br><u>Allergic reaction, n/N</u><br>IG: 1/47 vs. CG: 4/57	**Level of evidence**<br>3b↓<br><br>**Risk of bias**<br>Selection bias: –<br>Performance bias: –<br>Attrition bias: +<br>Detection bias: –<br><br>**Authors' conclusion**<br>"(in trauma cases with bleeding scalp injuries) Axiostat®, significantly reduced time to haemostasis and reduced blood loss during emergency and trauma as compared to conventional cotton gauze. Additionally, it is easy to apply and shows negligible side effects."

Study: Reference, aim, design, setting	Participants: selection criteria, characteristics	N Participants; Intervention (IG) vs. Control group (CG)	Main outcomes	Assessment: LoE, risk of bias; Conclusions
	endpoint or interfered with procedure and assessments in this study<br>• patients with a surgical/iatrogenic wound<br>• patients with a major head injury, spinal injury, neck injury, abdominal injury, deep wound injury, fracture, haemorrhagic shock, or foreign materials inside the wound<br>**Characteristics**<br><u>Age [y], mean ± SD</u><br>IG: 42.2 ± 11.7 vs. CG: 40.1 ± 12.8, p=0.41<br><u>Male, n (%)</u><br>IG: 32 (68) vs. CG: 41 (72), p=0.67<br><u>Wound duration, n (%)</u><br>*<1 hour* IG: 46 (97.9) vs. CG: 52 (91.2)<br>*1-3 hours* IG: 1 (2.1) vs. CG 5 (8.8), p=0.15			**Reviewers' conclusion**<br>The study results need to be interpreted with caution due to the small number of participants and risk of selection, performance and detection bias. Accuracy in the measurement of blood loss was doubtful. Patients did not fulfil the criteria of polytrauma (ISS≥15, multiple injuries).
**Winstanley (2019)**<br>"Catastrophic haemorrhage in military major trauma patients: a retrospective database analysis of haemostatic agents used on the battlefield". *J R Army Med Corps* 2019; 165 :405-409.<br>**Study design**<br>Comparative registry study	**Inclusion criteria**<br>• NISS ≥15<br>• injured in the Iraq or Afghanistan conflicts<br>**Exclusion criteria**<br>• NISS not reported<br>• multiple haemostatic agents used<br>**Characteristics**<br>98% blast and gunshot wounds<br><u>Age [y], mean ± SD</u><br>IG: 25.1 ± 7.6 vs. CG: 24.7 ± 8.6, p=0.52	**Participants**<br>N=3,792 patients<br>**Study groups**<br>IG: haemostatic agent used (N=317)<br>CG: no haemostatic agent used (N=3,475)<br><br>Hemcon and Quickclot were most commonly used, since 2010, the most common agent used has been Celox.	**Survival, all cases**<br><u>Survival, %</u><br>IG: 71.3 vs. CG: 64.0, p=0.01<br><u>Celox vs. no haemostatic agent, %-difference</u><br>14, p<0.00 (N=212 used Celox)<br><u>Quickclot vs. no haemostatic agent, %-difference</u><br>-6, p=0.63 (N=18 used Quickclot)<br><u>Hemcon vs. no haemostatic agent, %-difference</u><br>-8, p=0.13 (N=87 used Hemcon)<br>**Survival stratified by NISS**	Level of evidence<br>2b<br>Risk of bias<br>Selection bias: –<br>Performance bias: ?<br>Attrition bias: +<br>Detection bias: +<br><br>**Authors' conclusion**<br>"There is an association between the use of

Study: Reference, aim, design, setting	Participants: selection criteria, characteristics	N Participants; Intervention (IG) vs. Control group (CG)	Main outcomes	Assessment: LoE, risk of bias; Conclusions
(UK Joint Theatre Trauma Registry)<br><br>**Aim of the study**<br><br>"The aim of this study was to provide a descriptive analysis of the use of haemostatics in major trauma patients on the battlefield. We examined patient demographics, levels of injury severity and associated rates of survival."<br><br>**Setting**<br><br>UK military, Iraq / Afghanistan, 2003-2014	Male, n (%)<br><br>IG: 313 (99) vs. CG: 3,372 (97)<br><br>NISS, mean ± SD MD (95% CI)<br><br>IG: 43.4 ± 20.8 vs. CG: 42.4 ± 22.2, p=0.39		Celox vs. no haemostatic agent, %-difference<br><br>NISS 15–35: 6, p=0.28<br>NISS 36–55: 14, p=0.03<br>NISS 56–75: 24, p<0.00<br><br>Quickclot vs. no haemostatic agent, %-difference<br><br>NISS 15–35: 4, p=0.33<br>NISS 36–55: 9, p=1.00<br>NISS 56–75: −8, p=1.00<br><br>Hemcon vs. no haemostatic agent, %-difference<br><br>NISS 15–35: −11, p=0.42<br>NISS 36–55: 27, p=0.56<br>NISS 56–75: 1, p=0.13	haemostatic agents and improved survival, mostly in those with more severe injuries, which is particularly evident in those administered Celox."<br><br>**Reviewers' conclusion**<br><br>The study results need to be interpreted with caution due to the retrospective study design and risk of selection bias. The results are unadjusted and groups may differ regarding important unmeasured confounders.

+: low risk; –: high risk; ?: unclear risk; AIS: Abbreviated Injury Scale; CI: Confidence Interval; HR: Hazard Ratio; IQR: Interquartile Range; ISS: Injury Severity Score; ITT: Intention to Treat; MD: Mean Difference; MESS: Mangled Extremity Severity Score; NISS: new injury severity score; OR: Odds Ratio; RR: Relative Risk; SBP: Systolic blood pressure; SD: Standard Deviation; SEM: Standard Error of Mean; adj.: adjusted; d: days; g: grams, m: months; min: minute; y: years

Nasal balloon catheters

Study: Reference, aim, design, setting	Participants: selection criteria, characteristics	N Participants; Intervention (IG) vs. Control group (CG)	Main outcomes	Assessment: LoE, risk of bias; Conclusions
**García Callejo (2010)**<br><br>"Nasal packing in posterior epistaxis. Comparison of two methods". *Acta Otorrinolaringol Esp.* 2010; 61(3): 196–201.<br><br>**Study design**<br><br>Prospective cohort study	**Inclusion criteria**<br><br>• posterior epistaxis that required packing<br>• patients in whom conventional alternatives such as anterior packing with cotton, gauze or cellulose pads were ineffective in the first attempt or in repeated ER visits<br><br>**Exclusion criteria**	**Participants**<br><br>N=140 patients, 152 packings<br><br>**Study groups**<br><br>IG: Pneumatic packing device (105 packings in N=96 patients)<br><br>Pneumatic packing device with a length of 12cm coated in tetracaine paste with 2 chambers and anterior introduction; it accepts a maximum inflation with saline	**Unadjusted outcomes**<br><br>Control with a single packing<br><br>IG: 71 (67.6%) vs. CG: 37 (78.7%), p<0.001<br><br>Need for embolisation/ligation, n/N (%)<br><br>IG: 12 (11.4) vs. CG: 2 (4.2) , p<0.001<br><br>Need for RBC concentrate transfusion, n/N (%)<br><br>IG: 19/105 (18.1) vs. CG: 7/47 (14.8), p<0.001	**Level of evidence**<br><br>3b↓<br><br>**Risk of bias**<br><br>Selection bias: –<br><br>Performance bias: –<br><br>Attrition bias: +

Study: Reference, aim, design, setting	Participants: selection criteria, characteristics	N Participants; Intervention (IG) vs. Control group (CG)	Main outcomes	Assessment: LoE, risk of bias; Conclusions
**Aim of the study**<br>"The objective of this study was to assess the reliability of the two most commonly used types of posterior packing in terms of tolerance, comfort and capacity of terminating the haemorrhage."<br>**Setting**<br>Spain, 2003-2008	none reported<br>**Characteristics**[§]<br><u>Age [y], mean ± SD (range)</u><br>IG: 51.2 ± 11.8 (37-74)<br>CG: 54.8 ± 9.9 (40-68)<br><u>Male:female ratio</u><br>IG: 2.2:1<br>CG: 3.2:1<br>There were more cases assisted with pneumatic packing, with a statistically significant difference, among those patients *with clotting or platelet aggregation disorders*.<br>[§] p-value not reported	solution of 10 cc in the posterior compartment and up to 30 cc in the anterior. The intravenous preparation of the patient is similar to the control group.<br>CG: Classic posterior packing (47 packings in 44 patients)<br>Classic posterior packing with gauze soaked in tetracaine paste and impacted into the cavum and choana, introduced through the mouth using traction probe from the nostril involved. The packing is completed by adding gauze through the nostril until the maximum possible area of the nasal segment is filled. The patient is systematically administered 5 mg of diazepam and 2 g of metamizole intravenously 30 s before plugging.<br>Treatment assignment chosen by the doctor on duty accordance to the emergency considerations suggested by the situation in each case.	<u>Rebleeding with packing placed, n/N (%)</u><br>IG: 28/105 (26.6) vs. CG: 8/47 (17), p<0.001<br><u>Rebleeding just after removal, n/N (%)</u><br>IG: 11/105 (10.5) vs. CG: 1/47 (2.1), p<0.001<br><u>Pain during placement [VAS], mean ± SD</u><br>IG: 6.7 ± 1.7 vs. CG: 8.3 ± 1.5, p<0.001<br><u>Pain at 3rd day [VAS], mean ± SD</u><br>IG: 3.4 ± 2.2 vs. CG: 5.7 ± 2.7, p<0.001<br><u>Pain at removal [VAS], mean ± SD</u><br>IG: 1.3 ± 1.8 vs. CG: 2.1 ± 2.2, NS<br><u>Duration of placement [s], mean ± SD</u><br>IG: 36±19 vs. CG: 228 ± 102, p<0.001<br><u>Hospital stay [d], mean ± SD</u><br>IG: 5.2±1.3 vs. CG: 4.2±0.9, p<0.001<br><u>Definitive structural complications</u><br>IG: 8 (7.6) vs. CG: 2 (4.2), p<0.001	Detection bias: +<br>**Authors' conclusion**<br>"The classic posterior packing with gauze is less rapid and comfortable to adapt, but it ensures a higher success rate in the control of epistaxis, produces fewer local injuries and reduces health costs in comparison with inflatable balloon packing."<br>**Reviewers' conclusion**<br>The study results need to be interpreted with great caution due to the risk of selection and performance bias. Important confounders were not reported, and the analysis is unadjusted. Treatment was in-hospital, and epistaxis was due to trauma in less than 10% of patients, so that the results may not apply to the prehospital treatment of severely injured patients.
+: low risk; –: high risk; ?: unclear risk; ER: emergency room; NS: not significant; RBC: red blood cells; SD: Standard Deviation; VAS: visual analogue scale. adj.: adjusted; d: days; m: months; y: years				

Junctional bleeding

No studies identified.
